# Offensive unpredictability and defensive tracking in intergroup conflict show sex-differentiated patterns

**DOI:** 10.1016/j.isci.2026.117887

**Published:** 2026-10-15

**Authors:** Hejing Zhang, Churuo Zhang, Anastasia Widjaja, Yina Ma

**Affiliations:** 1 Bay Area School of Applied Psychological Sciences, Beijing Normal University, Zhuhai 519087, China; 2 Beijing Key Laboratory of Applied Experimental Psychology, National Demonstration Center for Experimental Psychology Education, Faculty of Psychology, Beijing Normal University, Beijing 100875, China; 3 Faculty of Psychology, Beijing Normal University, Beijing 100875, China; 4 Sino-Danish Center, University of Chinese Academy of Sciences, Beijing 100190, China; 5 CAS Key Laboratory of Behavioral Science, Institute of Psychology, Chinese Academy of Sciences, Beijing 100101, China; 6 IDG/McGovern Institute for Brain Research, Beijing Normal University, Beijing 100875, China; 7 State Key Laboratory of Cognitive Science and Mental Health, Institute of Psychology, Chinese Academy of Sciences, Beijing 100101, China

**Keywords:** intergroup conflict, sex difference, offense-defense dynamics, behavioral variability, stategic trackin

## Abstract

Intergroup conflict poses asymmetric strategic demands: acquiring resources through offense benefits from behavior that opponents cannot easily anticipate, whereas protecting resources through defense requires monitoring opponents and adjusting to threats. Analyzing behavioral data from 528 adults in a real-time offense-defense game, we found that offensive and defensive success were predicted by different decision patterns. Offensive success was associated with greater within-person contribution variability, a potential source of unpredictability, whereas defensive success was associated with stronger adaptive state-tracking of opponent behavior. These strategies aligned with sex-differentiated behavioral patterns: male participants showed greater variability and performed better in offense, whereas female participants showed stronger tracking and performed better in defense. Together, these findings identify dissociable behavioral strategies to success in offense and defense, offering a behavioral framework for understanding sex-linked variation in competitive social behavior.

## Highlights

• Unpredictability supports offensive success
• Opponent tracking supports defensive success
• Males show higher behavioral variability and outperform females in offense
• Females show stronger opponent-tracking and outperform males in defense

## Graphical abstract

**Figure:**
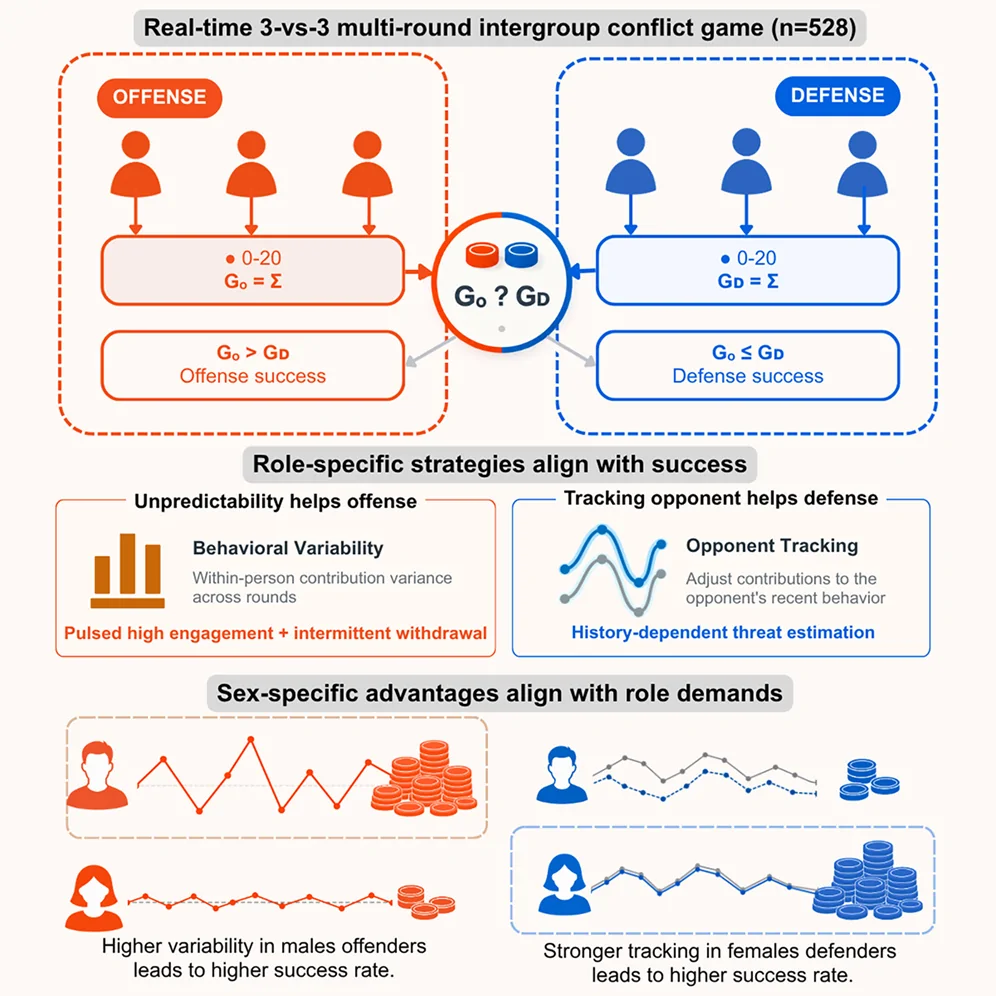

## Teaser

Behavioral neuroscience; Biological sciences; Natural sciences; Neuroscience

## Introduction

Intergroup conflict has acted as a powerful selective force throughout human evolution,^1^^,^^2^ shaping psychological and neurobiological systems for threat management, cooperation, and social competition.^3^^,^^4^ Critically, conflict places asymmetric survival demands on those who attack and those who defend: attackers seek to acquire resources by exploitation, whereas defenders aim to protect resources by monitoring and neutralizing threats.^4^ These distinct goals are thought to impose distinct computational demands.^5^^,^^6^^,^^7^ Across adversarial settings, successful attack often depends not only on the amount of force or resources invested, but also on limiting defenders’ ability to anticipate when, where, or how strongly exploitation will occur. Variability in offensive action can therefore make attacks harder to counter by preventing opponents from forming stable expectations about future moves.^8^ Defense, in contrast, requires continuous threat assessment and efficient allocation of protective effort: over-investment is costly, whereas under-investment leaves resources vulnerable, a logic formalized in attacker–defender models of strategic resource protection.^9^^,^^10^ This broader logic makes behavioral variability and opponent-contingent tracking theoretically meaningful candidate strategies for offensive and defensive roles, respectively. Although behavioral and game-theoretic models recognize this asymmetry,^11^^,^^12^ it remains unclear whether humans recruit functionally specialized cognitive and behavioral strategies to solve these distinct problems, and how such strategies are biologically regulated.

We propose that these role-specialized strategies may be consistent with sex-differentiated tendencies in risk sensitivity and social competition.^13^^,^^14^^,^^15^^,^^16^ Under sexual-selection and status-competition frameworks, male competition has often been discussed in relation to high-risk status contests, coalitionary aggression, and greater variance in reproductive or resource-related outcomes.^17^^,^^18^^,^^19^^,^^20^ Thus, males may, on average, be more likely to rely on high-stakes, variance-tolerant strategies necessary for maximizing status and resource acquisition.^21^^,^^22^ Females, in contrast, often bearing higher costs of injury and greater parental investment, may prioritize risk management and self-protection.^23^^,^^24^^,^^25^ Consistent with this view, female competition has been characterized as involving indirect, information-based strategies, including rival monitoring, reputational influence, selective information transmission, and coalitional gossip.^26^^,^^27^^,^^28^ These strategies may enhance sensitivity to changing social states and potential threats, supporting effective opponent tracking in defense. Thus, beyond simple differences in aggression,^4^^,^^15^ these ecological and social-competitive affordances may be associated with distinct strategic intuitions: biasing males toward generating unpredictability in offense, and females toward reactive adjustment in defense.

Beyond identifying the behavioral strategies associated with success in offense and defense, we also explored whether individual differences in salivary testosterone were related to these role-aligned behavioral patterns. Previous work suggested that testosterone-behavior associations vary across competitive contexts^29^^,^^30^ and may depend on which behaviors are useful for pursuing status or resources.^31^^,^^32^ We therefore asked, as a secondary exploratory question, whether within-sex variation in pre- and post-task salivary testosterone was related to behavioral variability and opponent-tracking across the offensive and defensive roles.

Here, we integrated behavioral, endocrine, and role-based analyses to examine offensive unpredictability and defensive tracking in intergroup conflict, and their associations with sex and salivary testosterone. We conducted a secondary analysis on a large-scale behavioral dataset^7^ with a real-time offender-defender conflict paradigm (*n* = 528, payoff matrix: Table S1; demographic descriptives: Table S2), significantly extending our original framework by integrating pre-task and post-task endogenous testosterone assessment (full details on saliva collection and assay in STAR Methods) with role-specific decision strategies. In this paradigm,^7^^,^^11^ six same-sex participants formed two competing three-person groups assigned to offensive and defensive roles. In each round, participants independently contributed tokens to their group pool; offense succeeded only by exceeding defense, whereas defense succeeded by matching or exceeding offense. This payoff asymmetry created two distinct optimization problems: offense required balancing contribution costs against opportunities for resource appropriation, whereas defense required estimating offensive threat to prevent loss without over-investing. We quantified two conflict-related strategies: behavioral unpredictability (indexing the dispersion and variability of trial-by-trial contributions within individuals^8^) and strategic tracking (indexing opponent-dependent updating, measured by the extent to which an individual’s contributions changed in response to opponents’ behavior across rounds^12^). We first tested whether behavioral variability and opponent-contingent tracking were differentially associated with success in offensive and defensive roles. We then examined whether these behavioral patterns differed by sex. Finally, in secondary, theory-guided endocrine analyses, we assessed whether pre-task and post-task salivary testosterone covaried with these behavioral patterns in sex- and role-specific ways.

## Results

### Offensive and defensive success rely on distinct behavioral strategies

To test whether humans recruit role-specialized cognitive strategies for conflict, we analyzed behavior in a real-time offender-defender game (*n* = 528). Teams of six same-sex participants were randomly assigned to asymmetric roles: three-person offense teams sought to acquire resources from an outgroup, while three-person defense teams sought to protect existing assets.^7^^,^^11^ The payoff structure imposed asymmetric risks (Table S1). Specifically, in each round, participants independently decided how many tokens to contribute to their group pool. If the offensive group’s total contribution exceeded that of the defensive group, the offense succeeded: offenders retained their own uninvested tokens and equally shared the defenders’ remaining uninvested tokens, whereas defenders earned zero for that round. If the defensive total matched or exceeded the offensive total, the attack failed and all participants retained only their own uninvested tokens. Thus, offense involved a trade-off between conserving one’s own endowment and investing enough to secure offensive success, whereas defense required safeguarding existing resources under a sharper threat of loss.

We quantified two strategy indices. Behavioral variability (indexing strategic unpredictability) was defined as the intra-individual variance in conflict-related contributions across 24 rounds (STAR Methods). Strategic tracking of opponents (indexing state estimation) was defined as the extent to which an individual’s contributions changed in response to the opponent’s previous actions (specifically, the *beta* coefficient derived from a rolling regression of opponent behavior; STAR Methods).

A robust complementary role-specific pattern emerged between these strategies and group outcomes. Behavioral variability interacted with role to predict group success (*Fisher’s z* = 6.389, *p* < 0.001). Specifically, variability was positively associated with success in offensive roles (*r*_*262*_ = 0.361, *p* < 0.001; Figure 1A), but negatively associated with success rate in defensive roles (*r*_*262*_ = −0.179, *p* = 0.003; Figure 1B), consistent with the idea that the same temporal pattern of contribution can have different functional consequences under asymmetric payoff structures. To confirm that this variability reflects a structured mixed-strategy for offensive success rather than random noise, we decomposed variability into engagement intensity (non-zero contribution level) and withdrawal frequency (zero contribution rate). High variability was driven by a “pulsed” pattern: combining high non-zero contribution level when engaging, interspersed with intermittent zero-contribution. Moreover, both engagement intensity (non-zero contribution level) (*r*_*246*_ = 0.284, *p* < 0.001) and zero-contribution rate (*r*_*262*_ = −0.589, *p* < 0.001) were independently associated with success in attackers. Although zero-contribution rate was negatively associated with success rate, it was systematically related to other task-relevant outcomes: it was associated with lower defensive resistance (defined as average contribution of defensive roles), r_262_ = −0.578, *p* < 0.001, greater average spoils on successful offensive rounds, r_241_ = 0.625, *p* < 0.001, and greater resource-extraction efficiency (calculated as spoils divided by offensive cost), r_241_ = 0.552, *p* < 0.001. This pattern argues against a pure noise account. If offensive variability merely reflected random fluctuation or poor control, it would not be expected to show systematic associations with defensive resistance and resource extraction. Instead, the results suggest a temporally pulsed mode of offensive engagement: larger active-round contributions were linked to successful attack, whereas intermittent zero-contribution rounds were associated with lower defensive investment and more efficient resource capture when offense succeeded. Thus, these results suggest that effective offensive strategies require a structured alternation between forceful engagement and strategic disengagement.

**Figure 1:**
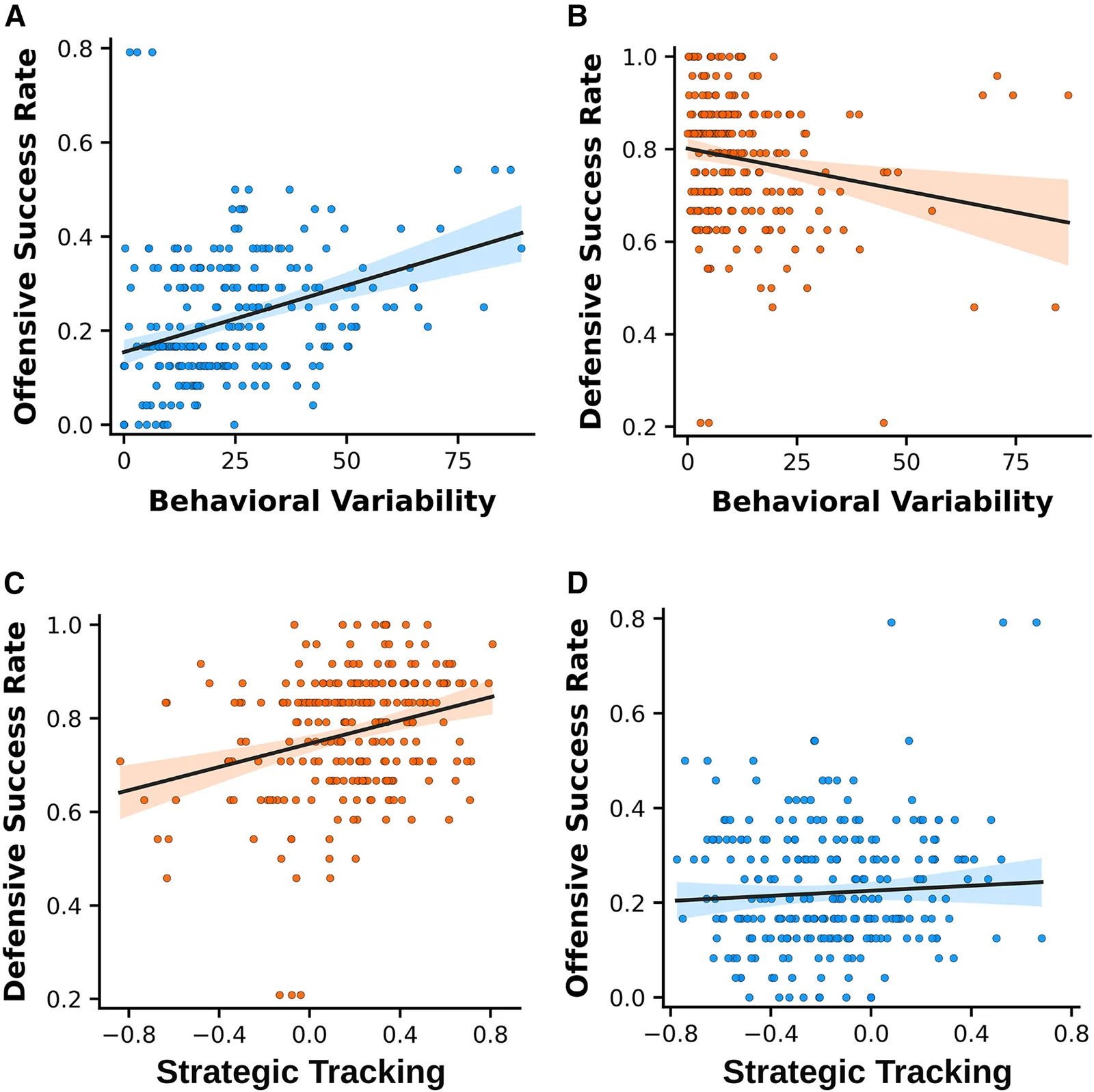
Offensive and defensive success rely on distinct behavioral strategies (A) Behavioral variability was positively associated with success in offensive roles (r_262_ = 0.361, *p* < 0.001, *n* = 264). (B) Behavioral variability was negatively associated with success in defensive roles (r_262_ = −0.179, *p* = 0.003, *n* = 264). (C) Strategic tracking of opponents was positively associated with success in defensive roles (r_249_ = 0.277, *p* < 0.001, *n* = 251). (D) Strategic tracking of opponents showed no significant association with success in offensive roles (r_262_ = 0.056, *p* = 0.365, *n* = 264). All associations were tested using two-tailed Pearson correlation tests. Each dot represents one participant. Behavioral measures were aggregated across 24 within-participant rounds. Solid lines show linear fits; shaded bands indicate 95% confidence intervals.

This confirms that unpredictability generation is an effective offensive policy (strategic unpredictability). In contrast, strategic tracking showed the complementary pattern (*Fisher’s z* = 2.578, *p* = 0.010). In defense, stronger tracking predicted higher success (*r*_*249*_ = 0.277, *p* < 0.001; Figure 1C), highlighting the adaptive value of monitoring opponent states. In offense, however, tracking was unrelated to success (*r*_*262*_ = 0.056, *p* = 0.365; Figure 1D). Together, these results confirm that success in intergroup conflict depends on role-congruent policies: unpredictability for offense, and state-tracking for defense.

### Sex differences map onto role-specific strategies

We next examined whether these strategic optima align with sex-differentiated behavioral priors. Males exhibited a general bias toward the pulsed, high-variability strategies. Across roles, males showed significantly higher behavioral variability than females (20.580 ± 1.010 vs. 15.510 ± 0.922; F_1, 524_ = 13.731, *p* < 0.001, partial η^2^ = 0.026, Figure 2A; full statistics in Table S4). This effect remained significant in a pooled single-slope ANCOVA controlling for opponent average contribution (F_1, 523_ = 9.902, *p* = 0.002, partial η^2^ = 0.019; full statistics in Table S5).

**Figure 2:**
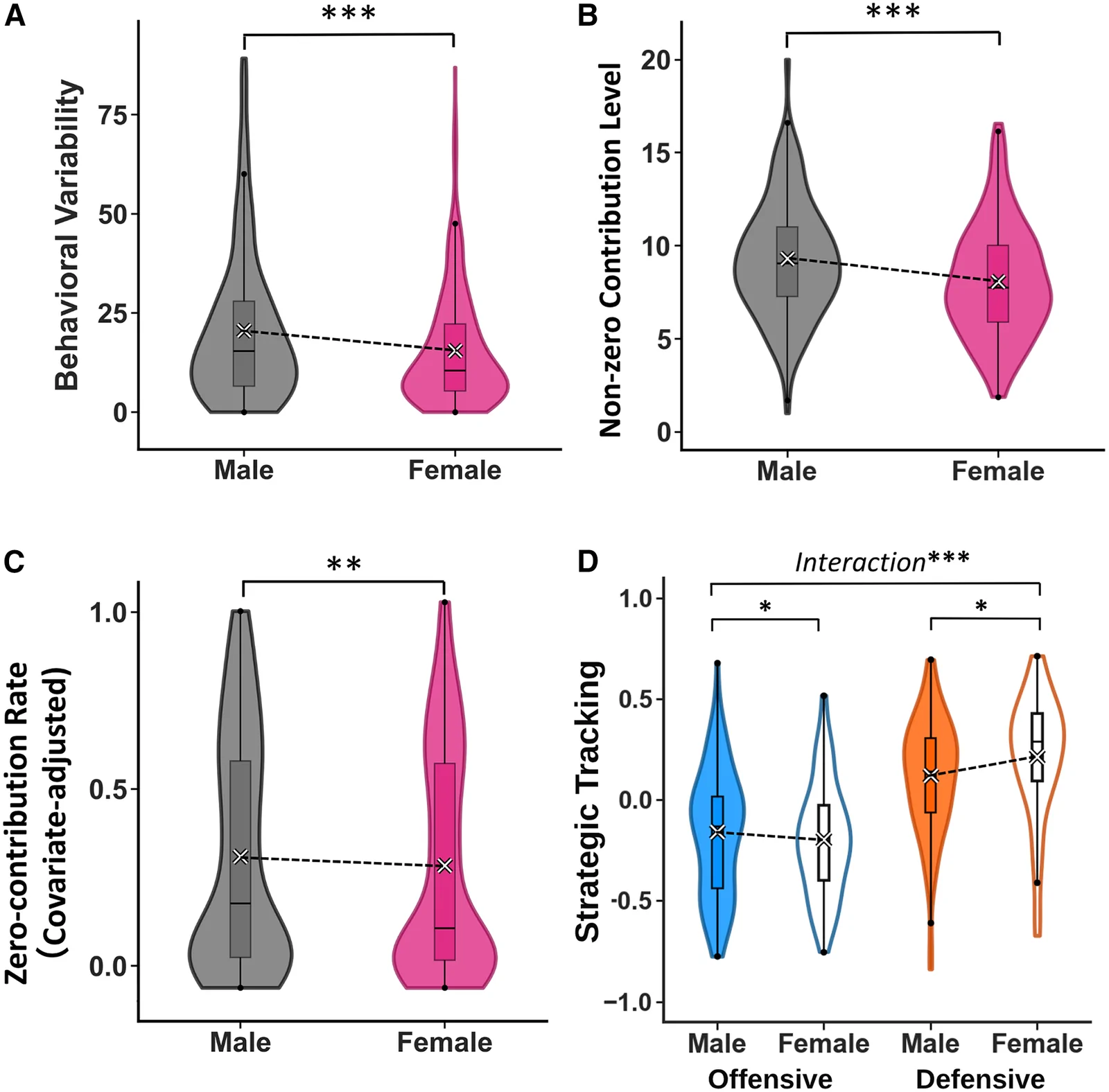
Sex differences in role-specific strategies (A) Males exhibited higher behavioral variability (20.580 ± 1.010 vs. 15.510 ± 0.922; F_1, 524_ = 13.731, *p* < 0.001, partial η^2^ = 0.026, *n* = 528). (B) Non-zero contribution level was higher in males than females (9.686 ± 0.212 vs. 8.272 ± 0.194; F_1, 508_ = 24.277, *p* < 0.001, partial η^2^ = 0.046, *n* = 512). (C) Zero-contribution rate did not differ significantly between males and females (0.312 ± 0.015 vs. 0.281 ± 0.014; F_1, 524_ = 2.299, *p* = 0.130, partial η^2^ = 0.004, *n* = 528). However, in the covariate-adjusted sensitivity analysis, males showed higher zero-contribution frequency than females after controlling for opponent average contribution (adjusted means: 0.324 ± 0.013 vs. 0.271 ± 0.012; F_1, 523_ = 8.466, *p* = 0.004, partial η^2^ = 0.016, *n* = 528). (D) Strategic tracking (opponent-contingent adjustment indexed by the coupling between one’s contributions and the opponent’s prior behavior) showed a significant sex × role interaction (F_1,511_ = 14.415, *p* < 0.001, partial η^2^ = 0.027, *n* = 515): females exhibited stronger positive tracking than males in defense (0.223 ± 0.025 vs. 0.120 ± 0.027; F_1, 511_ = 7.852, p_corr_ = 0.011, partial η^2^ = 0.015), and stronger negative tracking than males in offense (−0.200 ± 0.023 vs. −0.107 ± 0.028; F_1, 511_ = 6.577, p_corr_ = 0.021, partial η^2^ = 0.013). Violin plots show the distributions. Horizontal lines indicate medians, boxes span the interquartile range (25th–75th percentiles), crosses indicate means, and whiskers extend to 1.5 × the interquartile range beyond the box and are truncated at the observed minimum or maximum. Numerical values are group means ± SEM. Sex effects in A–C were tested using two-way ANOVAs with Sex and Role as between-subject factors; the sensitivity analysis in C used ANCOVA controlling for opponent average contribution. The Sex × Role interaction in D was tested using the same two-way ANOVA, followed by Bonferroni-adjusted comparisons between males and females within each role. All *n* values denote the number of participants contributing valid data to the corresponding analyses; measures were aggregated across 24 within-participant rounds. p_corr_ values are Bonferroni-adjusted *p* values for comparisons between males and females within each role. ∗*p* < 0.05, ∗∗*p* < 0.01, and ∗∗∗*p* < 0.001.

To clarify the behavioral components underlying this variability effect, we decomposed variability into non-zero contribution level and zero-contribution rate. Compared with females, males showed significantly higher non-zero contribution level in the primary ANOVA (9.686 ± 0.212 vs. 8.272 ± 0.194; F_1, 508_ = 24.277, *p* < 0.001, partial η^2^ = 0.046, Figure 2B), and this sex effect remained significant after controlling for opponent average contribution (F_1, 507_ = 19.748, *p* < 0.001, partial η^2^ = 0.037). For zero-contribution frequency, the primary ANOVA did not show a significant sex difference (0.312 ± 0.015 vs. 0.281 ± 0.014; F_1, 524_ = 2.299, *p* = 0.130, partial η^2^ = 0.004). However, in the covariate-adjusted sensitivity analysis, males showed higher zero-contribution frequency than females after controlling for opponent average contribution (adjusted means: 0.324 ± 0.013 vs. 0.271 ± 0.012; F_1, 523_ = 8.466, *p* = 0.004, partial η^2^ = 0.016, Figure 2C). This pattern suggests that the male strategy is characterized by pulsed, high-intensity engagement, which generates unpredictability, rather than simple differences in effort.

Females, in contrast, exhibited a role-specific bias toward adaptive tracking. A significant role-by-sex interaction emerged for the tracking index (*F*_*1,511*_ = 14.415, *p* < 0.001, partial η^2^ = 0.027, Figure 2D). In the defensive role, female defenders showed significantly stronger positive opponent-contingent tracking than male defenders (0.223 ± 0.025 vs. 0.120 ± 0.027; F_1, 511_ = 7.852, *p*_corr_ = 0.011, partial η^2^ = 0.015). In the offensive role, female offenders showed more negative opponent-contingent coupling than male offenders (−0.200 ± 0.023 vs. −0.107 ± 0.028; F_1, 511_ = 6.577, *p*_corr_ = 0.021, partial η^2^ = 0.013). This interaction remained significant after controlling for opponent average contribution in the pooled single-slope ANCOVA, F_1, 510_ = 14.353, *p* < 0.001, partial η^2^ = 0.027. The covariate-adjusted simple effects showed the same pattern in defense (female vs. male: 0.218 ± 0.026 vs. 0.117 ± 0.028; F_1, 510_ = 7.472, *p*_corr_ = 0.013, partial η^2^ = 0.014) and offense (female vs. male: −0.197 ± 0.025 vs. −0.102 ± 0.028; F_1, 510_ = 6.770, *p*_corr_ = 0.019, partial η^2^ = 0.013). Thus, females showed stronger opponent-contingent adjustment than males in both roles, but this adjustment was expressed in opposite directions: positive tracking in defense and more negative coupling in offense.

### Males excel in offense while females excel in defense

Given the alignment between strategy and sex, we examined whether these biases translate into performance asymmetries. We analyzed four complementary indices: outcomes (success rate, earnings) and decision process metrics (decision rationality indexed by the average deviation from the best response given teammate and opponent behavior,^33^^,^^34^ response time). Decision rationality (STAR Methods) indexed the negative deviation from the estimated best response, which was calculated from teammates’ and opponents’ immediately preceding-round contributions. Higher values, i.e., values closer to zero, indicate smaller deviations from the estimated best response and therefore greater rationality.

A consistent sex × role interaction emerged across all metrics (success rate: *F*_*1,524*_ = 35.103, *p* < 0.001, partial η^2^ = 0.063, Figure 3A; earnings: *F*_*1,524*_ = 7.806, *p* = 0.005, partial η^2^ = 0.015, Figure 3B; decision rationality: *F*_*1,524*_ = 15.751, *p* < 0.001, partial η^2^ = 0.029, Figure 3C; response time: *F*_*1,524*_ = 3.974, *p* = 0.047, partial η^2^ = 0.008, Figure 3D). These sex-by-role performance patterns were retained after accounting for the opponent behavioral environment (success rate: *F*_*1,523*_ = 37.015, *p* < 0.001, partial η^2^ = 0.066; earnings: *F*_*1,523*_ = 24.940, *p* < 0.001, partial η^2^ = 0.046; decision rationality: *F*_*1,523*_ = 15.721, *p* < 0.001, partial η^2^ = 0.029; response time: *F*_*1,523*_ = 4.109, *p* = 0.043, partial η^2^ = 0.008).

**Figure 3:**
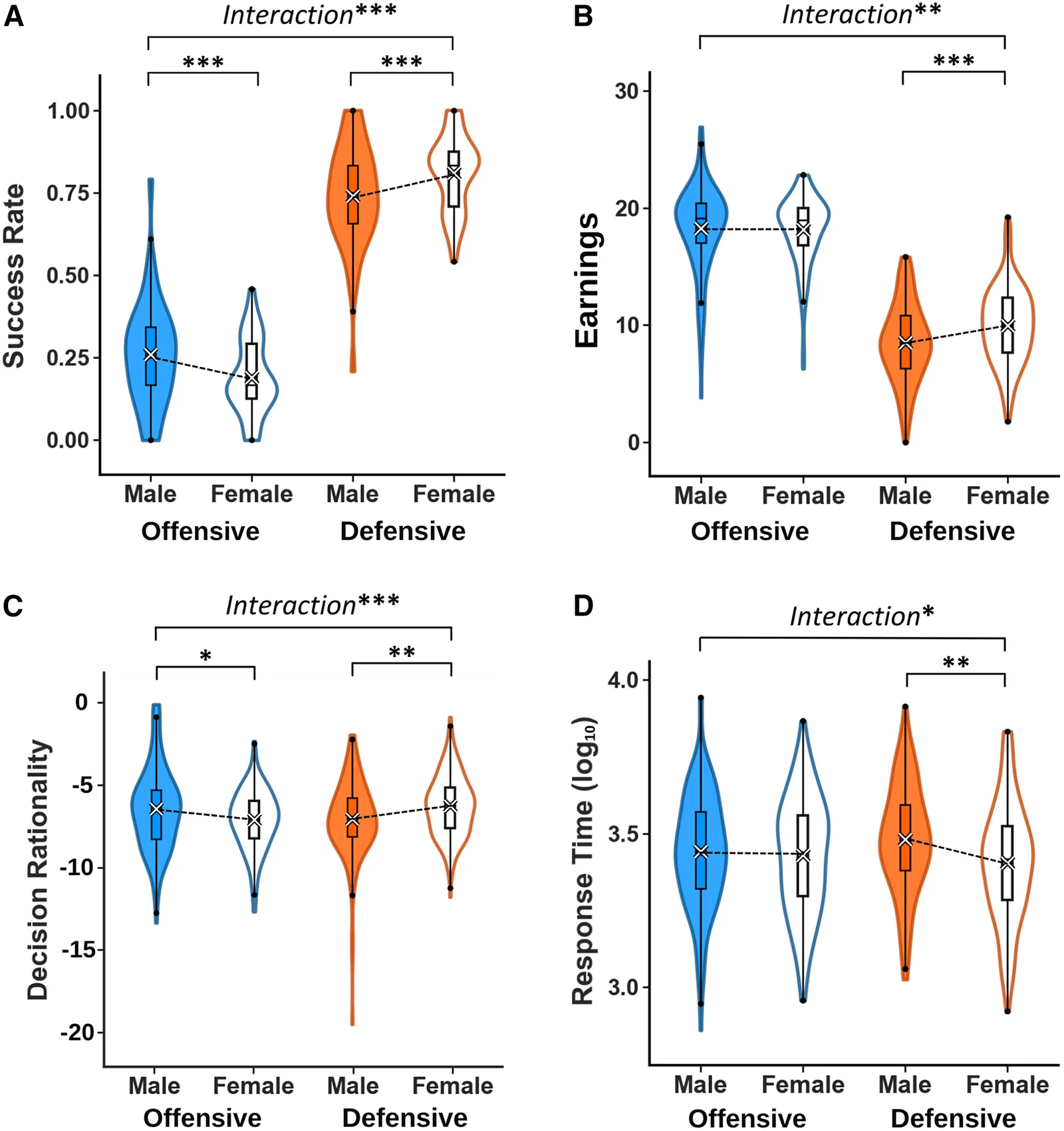
Sex-differentiated performance advantages in offensive and defensive roles (A) Success Rate. A significant interaction between role and sex was observed (interaction: F_1,524_ = 35.103, *p* < 0.001, partial η^2^ = 0.063), indicating that males achieved higher success rates than females in offensive roles (Male vs. Female: 0.259 ± 0.014 vs. 0.189 ± 0.009; F_1, 524_ = 17.551, p_corr_ < 0.001, partial η^2^ = 0.032), whereas females outperformed males in defensive roles (Female vs. Male: 0.811 ± 0.009 vs. 0.741 ± 0.014; F_1, 524_ = 17.551, p_corr_ < 0.001, partial η^2^ = 0.032). (B) Earnings. Role and sex also showed a significant interaction in earnings (Interaction: F_1,524_ = 7.806, *p* = 0.005, partial η^2^ = 0.015). No significant sex difference was observed in offensive earnings (male vs. female: 18.281 ± 0.339 vs. 18.159 ± 0.246; F_1,524_ = 0.088, p_corr_ = 1.000, partial η^2^ = 0.000), whereas in defensive roles females earned significantly more than males (Female vs. Male: 9.985 ± 0.273 vs. 8.485 ± 0.309; F_1, 524_ = 13.355, p_corr_ < 0.001, partial η^2^ = 0.025). (C) Decision rationality. Decision rationality measured as the negative absolute deviation from the estimated best response (higher values, i.e., less negative scores, indicate greater rationality), differed significantly across sex and role (Interaction: F_1,524_ = 15.751, *p* < 0.001, partial η^2^ = 0.029). Males demonstrated more rational decisions in offensive roles (male vs. female: −6.433 ± 0.234 vs. −7.124 ± 0.154; F_1, 524_ = 6.589, p_corr_ = 0.021, partial η^2^ = 0.012), while females outperformed males in defensive roles (Female vs. Male: −6.257 ± 0.158 vs. −7.077 ± 0.223; F_1, 524_ = 9.276, p_corr_ = 0.005, partial η^2^ = 0.017). (D) Response time. Response time exhibited a significant interaction between role and sex (Interaction: F_1,524_ = 3.974, *p* = 0.047, partial η^2^ = 0.008), suggesting a role-specific difference in decision-making speed. Female defenders responded significantly faster than male defenders (Female vs. Male: 3.405 ± 0.016 vs. 3.481 ± 0.017; F_1, 524_ = 10.985, p_corr_ = 0.002, partial η^2^ = 0.021), while no significant sex difference was observed in offensive roles (F_1,524_ = 0.245, p_corr_ = 1.000, partial η^2^ = 0.000). Violin plots show the distributions. Horizontal lines indicate medians, boxes span the interquartile range (25th–75th percentiles), crosses indicate means, and whiskers extend to 1.5 × the interquartile range beyond the box and are truncated at the observed minimum or maximum. Numerical values are group means ± SEM. All analyses used two-way ANOVAs with Sex and Role as between-subject factors, followed by Bonferroni-adjusted comparisons between males and females within each role. For all images, *n* = 528 participants. Sucess rate, earnings, and response time were summarized across 24 rounds; decision rationality was averaged across 2-24 rounds. p_corr_ values are Bonferroni-adjusted *p* values for comparisons between males and females within each role. ∗*p* < 0.05, ∗∗*p* < 0.01, and ∗∗∗*p* < 0.001.

Males outperformed females in offensive roles: they achieved higher success rates (0.259 ± 0.014 vs. 0.189 ± 0.009; F_1, 524_ = 17.551, *p*_*corr*_ < 0.001, partial η^2^ = 0.032, Figure 3A), and made decisions that were closer to the theoretical optimum (−6.433 ± 0.234 vs. −7.124 ± 0.154; F_1, 524_ = 6.589, *p*_*corr*_ = 0.021, partial η^2^ = 0.012, Figure 3C), whereas the offensive earnings difference was not significant (18.281 ± 0.339 vs. 18.159 ± 0.246; F_1,524_ = 0.088, *p*_*corr*_ = 1.000, partial η^2^ = 0.000, Figure 3B). In supplementary pooled single-slope ANCOVAs, the same offensive pattern remained for success rate (0.281 ± 0.013 vs. 0.204 ± 0.011; F_1,523_ = 22.336, *p*_*corr*_ < 0.001, partial η^2^ = 0.041) and decision rationality (−6.427 ± 0.210 vs. −7.120 ± 0.187; F_1,523_ = 6.574, *p*_*corr*_ = 0.021, partial η^2^ = 0.012), and also emerged for earnings (20.189 ± 0.179 vs. 19.436 ± 0.159; F_1,523_ = 10.680, *p*_*corr*_ = 0.002, partial η^2^ = 0.020).

In contrast, females outperformed males in defensive roles. Female defenders achieved significantly higher success rates (0.811 ± 0.009 vs. 0.741 ± 0.014; F_1, 524_ = 17.551, *p*_*corr*_ < 0.001, partial η^2^ = 0.032, Figure 3A) and earned substantially more (9.985 ± 0.273 vs. 8.485 ± 0.309; F_1, 524_ = 13.355, *p*_*corr*_ < 0.001, partial η^2^ = 0.025, Figure 3B) than male defenders. Crucially, process measures indicated that this advantage was underpinned by greater cognitive efficiency: female defenders not only made more rational decisions (−6.257 ± 0.158 vs. −7.077 ± 0.223; F_1, 524_ = 9.276, *p*_*corr*_ = 0.005, partial η^2^ = 0.017, Figure 3C) but also responded significantly faster than male defenders (3.405 ± 0.016 vs. 3.481 ± 0.017; F_1, 524_ = 10.985, *p*_*corr*_ = 0.002, partial η^2^ = 0.021, Figure 3D). These defensive advantages remained in the supplementary pooled single-slope ANCOVAs (success rate: 0.790 ± 0.012 vs. 0.727 ± 0.012; F_1,523_ = 14.821, *p*_*corr*_ < 0.001, partial η^2^ = 0.028; earnings: 8.134 ± 0.164 vs. 7.265 ± 0.173; F_1,523_ = 14.224, *p*_*corr*_ < 0.001, partial η^2^ = 0.026; decision rationality: −6.263 ± 0.193 vs. −7.081 ± 0.203; F_1,523_ = 9.151, *p*_*corr*_ = 0.005, partial η^2^ = 0.017; response time: 3.428 ± 0.016 vs. 3.497 ± 0.017; F_1,523_ = 9.064, *p*_*corr*_ = 0.005, partial η^2^ = 0.017), suggesting that defensive tracking is a more fluent, accessible heuristic for females.

### Exploratory associations between salivary testosterone and role-aligned behavior

As a secondary exploratory analysis, we examined whether individual differences in salivary testosterone were related to the role-aligned behavioral patterns identified above. Because this question concerned variation among individuals within each sex, rather than differences in mean testosterone levels between men and women, all analyses were conducted separately for men and women. The pre-task and post-task values showed substantial cross-time consistency in individual ranking within both men and women, and this relationship remained after adjusting for assay plate, testing time, age, and behavioral role (Table S11 and Methods S1).

In male offenders, higher log-transformed testosterone values were associated with greater behavioral variability (pre-task: r_108_ = 0.225, *p* = 0.018; post-task: r_108_ = 0.287, *p* = 0.002, Figure S2A), with post-task testosterone also covarying with offensive success (r_108_ = 0.207, *p* = 0.030, Figure S2B). In female defenders but not female offenders, higher salivary testosterone values were associated with stronger strategic tracking (pre-task: r_103_ = 0.224, *p* = 0.022; post-task: r_103_ = 0.345, *p* < 0.001, Figure S2C and Table S6). These findings are presented as preliminary associations between individual differences in salivary testosterone and behavior within each sex, rather than as tests of male-female differences in testosterone levels. Full descriptive statistics are reported in Table S3; unadjusted, baseline-adjusted, and between-role association analyses in Table S6; testing-time-adjusted associations in Table S10; cross-time consistency analyses in Table S11; and focal post-task associations in Figure S2.

## Discussion

Intergroup conflict is often conceptualized as a unitary struggle for dominance,^4^^,^^35^^,^^36^ yet our findings reveal that it relies on a dissociated strategy architecture. We report that offensive and defensive success are underpinned by opposing behavioral patterns. Offense, the act of resource acquisition,^4^^,^^8^ depends on strategic unpredictability—a “pulsed”, high-variability pattern of engagement that prevents opponents from anticipating attacks. Defense, the act of resource preservation,^4^^,^^5^ depends on adaptive state-tracking—a history-dependent, vigilance-oriented strategy that continuously estimates opponent threat levels. Crucially, these strategies are not randomly distributed but align with sex-differentiated tendencies: males exhibit a generalized bias toward the unpredictable policies effective in offense, whereas females exhibit a context-specific bias toward the tracking policies effective in defense. Furthermore, salivary testosterone covaried with circumscribed role-congruent strategies, i.e., offensive variability in male attackers and defensive tracking in female defenders, across pre-task, post-task, and baseline-adjusted post-task analyses. These conclusions are grounded in an asymmetric payoff structure that distills offense and defense into their core strategic demands: acquiring resources under uncertainty and protecting resources under threat.

Why do sexes diverge along these specific strategic lines? We interpret these patterns as average, role-congruent tendencies under the asymmetric demands of this task, rather than fixed sex-specific specializations. Both males and females can engage in offense and defense, but the two roles entail distinct strategic demands that may align differently with sex-associated tendencies in risk tolerance, threat sensitivity, and information use. Males, who have historically faced a higher variance in reproductive payoffs,^19^^,^^37^ appear to encode a “high-volatility prior”, enduring the costs of variable engagement to maximize the probability of high-stakes returns. Females, on the other hand, incurring higher costs of injury and parental investment^23^^,^^25^ appear to encode a “precision prior”, prioritizing error minimization and sensitivity to social cues. This account also accommodates the possibility that female competition is active and strategic rather than passive: work on competitive gossip and informational competition emphasizes monitoring rivals, updating socially relevant information, and using such information to manage competitive threats.^26^ Such information-sensitive strategies may fit the defensive demands of the present task, where success depended on tracking opponent behavior and adjusting contributions accordingly. Importantly, our data indicate that these sex-differentiated priors reflect functional competence. The male advantage in offense and the female advantage in defense were accompanied by higher decision rationality (decisions closer to the theoretical best response^33^^,^^34^) and greater economic efficiency (earned more). This implies that the male tendency toward variability is not merely impulsivity, but a potentially functional tactic to capitalize on uncertainty, while the female tendency toward tracking may reflect an efficient heuristic for threat management. Thus, these sex-linked tendencies may be understood as role-congruent heuristics supporting behavioral adaptation in this intergroup contest. The present data cannot determine whether these tendencies reflect evolved affordances, contemporary gendered socialization, prior competitive experience, or their interaction.^38^^,^^39^

The exploratory testosterone analyses provide a preliminary link between endocrine variation and the behavioral strategies identified in the task. Within each sex, salivary testosterone covaried with the strategy most closely associated with success in the corresponding role: offensive variability in male attackers and defensive tracking in female defenders. This pattern fits context-dependent accounts in which testosterone is linked not simply to generalized aggression, but to behaviors that support competitive goals in a given setting.^29^^,^^31^^,^^40^ In the present task, the relevant behaviors differed because offense involved acquiring resources, whereas defense involved protecting them. These findings should nevertheless be interpreted cautiously because estimates from cotton-collected salivary immunoassays can depend on both the collection procedure and the analytical method, particularly at the lower testosterone concentrations typically observed in women.^41^^,^^42^^,^^43^^,^^44^ Replication using more precise serum-based testosterone measures will be needed to establish the robustness of these associations, while placebo-controlled testosterone-administration studies would provide a more direct test of causality.^29^^,^^43^^,^^44^

In conclusion, human intergroup competition is shaped by the asymmetric demands of acquiring and protecting resources. Our findings identify distinct, role-aligned behavioral strategies for meeting these demands: greater variability was associated with offensive success, whereas stronger opponent tracking was associated with defensive success. The expression of these strategies also differed by sex, providing a framework for understanding sex- and role-related variation in intergroup decision-making.

### Limitations of the study

Several limitations warrant consideration. First, this study represents a secondary analysis^7^ combining behavioral data with endogenous testosterone assessment. Because the testosterone hypotheses and analyses were not preregistered, and testosterone was assessed only before and after the task in a correlational design, these findings should be treated as exploratory and require replication. This two-time-point design cannot characterize the full trajectory of testosterone change or separate baseline hormonal differences from challenge-related responses; future studies should address this issue with denser sampling and explicit testosterone-change models. Whether testosterone causally alters offensive variability or defensive tracking awaits direct experimental tests.^45^^,^^46^ Second, the cognitive origin of offensive variability remains to be specified. It is unclear whether the high-variability offense strategy represents a deliberate, top-down calculation to prevent prediction (as game theory implies^47^) or a biological heuristic.^48^^,^^49^ Future work using cognitive probing could clarify whether these patterns arise from explicit planning or implicit priors. Third, our design involved interactions between same-sex groups (i.e., male-vs-male or female-vs-female). This isolates baseline sex-specific priors but leaves open how strategies adapt in cross-sex contexts. Although we statistically controlled for opponents’ average contribution, such adjustment cannot fully separate an individual’s strategy from the reciprocal dynamics that unfold during interaction. A key question for future research is how these distinct priors interact in direct competition,^16^^,^^50^ specifically, whether the high-variability offense of males overwhelms the tracking capacity of female defenders, or whether female vigilance effectively decodes and neutralizes that variability.

## Resource availability

### Lead contact

Further information and requests for resources should be directed to and will be fulfilled by the lead contact, Yina Ma (yma@bnu.edu.cn).

### Materials availability

This study did not generate new, unique reagents.

### Data and code availability

• Data: The de-identified behavioral and hormonal datasets supporting the findings of this study, together with figure source data, are publicly available on the Open Science Framework (OSF): https://doi.org/10.17605/OSF.IO/C5FWE.
• Code: SPSS syntax files and plotting code used for the analyses reported in this paper are publicly available on OSF: https://doi.org/10.17605/OSF.IO/C5FWE.
• Other items: Participant task instructions are publicly available on OSF at the repository above. Any additional information or materials required to reanalyze the data reported in this paper are available from the lead contact upon request.

## Acknowledgments

This work was supported by Brain Science and Brain-like Intelligence Technology-National Science and Technology Major Project (2022ZD0211000 to Y. M.), the 10.13039/501100001809National Natural Science Foundation of China (Projects 32125019, 32430041 to Y. M.; Projects 32300867 to H. Z.), Guangdong Provincial Educational Science Planning Project (2023GXJK6670 to H. Z.), and 10.13039/501100002726Beijing Normal University at Zhuhai Research Startup Project (12900-312200502507 to H. Z.).

## Author contributions

Y.M. and H.Z. conceived the project and designed the experiment; H.Z. implemented the experiment and collected data; H.Z. and C.Z. analyzed the data under the supervision of Y.M.; Y.M. and H.Z. interpreted the results; A.W., H.Z., and C.Z. prepared the figures; Y.M. and H.Z. wrote the original and final versions of the manuscript. All authors approved the final version of the manuscript for submission.

## Declaration of interests

The authors declare no competing interests.

## Declaration of generative AI and AI-assisted technologies in the manuscript preparation process

During the preparation of this work, the authors used ChatGPT (OpenAI) to assist with literature search and language refinement. After using this tool, the authors reviewed and edited the content as needed and take full responsibility for the content of the publication.

## STAR★Methods

### Key resources table

**Table:** 

REAGENT or RESOURCE	SOURCE	IDENTIFIER
**Commercial assays or kits**		

Salivary Testosterone ELISA Kit	DRG Instruments GmbH, Germany	Cat. No. SLV-3013

**Software and algorithms**		

IBM SPSS Statistics	IBM Corp.	Version 27.0.1.0
Python	Python Software Foundation	Version 3.8.20

**Deposited data**		

Data and analysis materials	Open Science Framework	https://doi.org/10.17605/OSF.IO/C5FWE

### Experimental model and study participant details

#### Participants

We analyzed data from 528 healthy participants (mean age = 22.10 ± 0.11 years; 240 males, 288 females; see Table S2 for demographic details): 120 male offenders (mean age = 22.60 ± 0.26), 120 male defenders (mean age = 22.84 ± 0.25), 144 female offenders (mean age = 21.76 ± 0.20), and 144 female defenders (mean age = 21.42 ± 0.20). All participants had normal or corrected-to-normal vision and reported no history of psychiatric or neurological disorders. Individuals with academic training in psychology or economics were excluded before participation to minimize prior strategic knowledge. No deception was involved. Written informed consent was obtained following full disclosure of the procedures and acknowledgment of the right to withdraw at any point. The study protocol was approved by the Institutional Review Board at the State Key Laboratory of Cognitive Neuroscience and Learning, Beijing Normal University, China (Protocol IORG0004944), and was conducted in accordance with the Declaration of Helsinki.

##### Note on secondary analysis

This dataset is part of a larger project on the neurobiology of conflict.^7^ While previous work focused on neural synchronization, the current study uniquely examines endogenous testosterone and its relation to individual decision strategies, which were not analyzed in prior reports. Although salivary testosterone was collected in the original study, the present sex- and testosterone-related analyses were not preregistered and are therefore treated as secondary, theory-guided analyses rather than confirmatory tests.

##### Data exclusion and final sample

The initial cohort comprised 528 participants.^7^ No behavioral exclusions were applied; therefore, the primary behavioral analyses were conducted in the full behavioral sample of *n* = 528. These analyses included the strategy–outcome analyses, sex-difference analyses, and sex-by-role performance analyses. Analyses of specific derived behavioral indices used all available valid estimates for the corresponding index. Therefore, degrees of freedom vary slightly across analyses when a given index could not be computed for all participants. For example, analyses involving active-contribution magnitude or strategic tracking included only participants with valid estimates for those derived indices. Descriptive statistics for the full behavioral sample are reported in Table S2.

Testosterone-related analyses were conducted in the endocrine analytic sample, selected from the full sample by retaining participants whose pre-task and post-task testosterone concentrations fell within the manufacturer-specified analytical range of the DRG Salivary Testosterone ELISA kit (SLV-3013; 0.94–1000 pg/mL). Of the original 528 participants, 47 were excluded from the endocrine analyses because one or both saliva samples were insufficient, degraded, or otherwise unsuitable for assay. An additional 24 participants were excluded because at least one of their two salivary testosterone measurements fell outside the manufacturer-specified analytical range. The final endocrine analytic sample therefore included 457 participants: 110 male offenders, 112 male defenders, 124 female offenders, and 111 female defenders. Descriptive statistics for the endocrine analytic sample are also reported in Table S2, and the ranges of pre-task and post-task salivary testosterone concentrations are presented in Figure S1.

The endocrine screening criterion was used only to define the testosterone analytic sample and was not applied to the primary behavioral analyses. To evaluate whether the behavioral findings were affected by this sample definition, we repeated the primary behavioral analyses in the 457-participant endocrine analytic sample. The same substantive pattern of results was observed, indicating that the behavioral conclusions were stable across the full behavioral sample and the endocrine analytic sample (Figures S3–S5; Tables S7 and S8).

### Method details

#### Intergroup conflict task

Participants were organized into 6-person groups (same-sex) and randomly assigned to two competing subgroups: attacker group (offenders, *n* = 3) and defender group (defenders, *n* = 3). After a practice session, participants completed a formal decision-making session consisting of 24 rounds of real-time resource competition with full resets between rounds (i.e., payoffs did not accumulate across rounds). The task involved real monetary incentives and no deception. Participants received a fixed show-up fee of RMB 80 plus a performance-based bonus. After task completion, two of the 24 rounds were randomly selected, and earnings from those rounds were converted into RMB at a 1:1 token-to-RMB exchange rate. This incentive scheme ensured that each round was potentially payoff-relevant while minimizing cumulative payoff effects and time-dependent strategic carryover that might arise from summing or averaging earnings across all rounds.

The intergroup contest was a dynamic, fully incentivized resource competition between offender and defender groups, featuring an asymmetric payoff structure. The round-level payoff contingencies are summarized in Table S1. At the start of each round, participants had 12 s to decide how many tokens (0–20) to contribute to their group pool. Decisions were made independently without within-team communication. Following the decision phase, a jittered delay (6–10 s) preceded a 10-s outcome screen, which displayed: (i) individual in-group contributions, (ii) total group contributions for offense (G_O_) and defense (G_D_), (iii) the round outcome, and (iv) one’s own earning. Although direct communication was prohibited, the feedback provided participants with trial-by-trial information about both teammates and opponents. This information structure allowed participants to monitor others’ behavior over time and supported strategic adjustment in subsequent decisions.

Outcomes were determined by the relative magnitude of group contributions. (i) Offensive victory (G_O_ > G_D_): If the offensive total exceeded the defensive total, the attack succeeded. Offenders retained their uninvested tokens and equally divided the defenders’ unspent tokens (“spoils”). Defenders received zero payoff for the round. (ii) Defensive victory (G_O_ ≤ G_D_): If the defensive total equaled or exceeded the offensive total, the attack failed. All participants (offenders and defenders) retained only their initial uninvested tokens; no resource transfer occurred.

To minimize demand characteristics and avoid explicit moral framing, the instructions avoided terms such as “attack,” “defense,” “offense,” and “protection.” Participants instead received neutral descriptions of the task structure and payoff contingencies, allowing the strategic meaning of each assigned position to be inferred from the payoff structure rather than from explicit verbal labels. This payoff matrix created functionally distinct strategic situations for the two roles. Participants in the defensive role faced a stronger common-fate structure and a more severe cost of failure, because unsuccessful defense resulted in complete loss of round earnings. By contrast, participants in the offensive role could still retain their own uninvested tokens even when offense failed, creating a more asymmetric risk-reward structure. Thus, the distinction between offense and defense was implemented through the incentive structure of the game rather than through explicit wording.

#### Salivary testosterone assessment

##### Sample collection

Saliva samples were collected at two time points: before the 24-round conflict task and immediately after task completion. Before the first sample, participants were instructed to refrain from eating, drinking, smoking, tooth brushing, and vigorous physical activity for 60 min, and to rinse their mouth with water 30 min before collection. These restrictions were maintained throughout the experimental session until the post-task sample was obtained. Samples were collected using a sterile cotton swab placed under the tongue for approximately 5 min without chewing. After collection, samples were sealed, labeled, and stored at −80°C until assay.

##### Assay protocol

Prior to assay, samples were thawed and centrifuged at 4°C (3000 g, 10 min). Testosterone concentrations were quantified using a commercial enzyme-linked immunosorbent assay (ELISA) kit (DRG Instruments GmbH, Germany; Cat. No. SLV-3013). All assays were performed by the BGI Proteomics Testing Center (Beijing) following the manufacturer’s protocol.

##### Time-of-day robustness

Given the broad diurnal window of saliva collection (08:00–19:00), time of day was included as a covariate in sensitivity analyses to account for potential diurnal variation in testosterone concentrations. Full results from these adjusted models are reported in Tables S9 and S10.

### Quantification and statistical analysis

#### Quantification of decision strategies

To characterize individual decision-making patterns, we calculated two primary strategic indices based on trial-by-trial contribution data.

##### Behavioral variability

We defined behavioral variability as the intra-individual variance of contributions across the 24 rounds. Higher values indicate a wider dynamic range of engagement, effectively increasing unpredictability.

To determine whether variability reflected random noise or a structured strategy, we further decomposed contribution behavior into two sub-components: (i) non-zero contribution level: The mean contribution amount calculated only from rounds where the participant chose to contribute (i.e., contribution >0). This captures the magnitude of “strikes”; (ii) zero-contribution rate: The proportion of rounds in which the participant contributed zero tokens. This captures the tendency for intermittent disengagement.

##### Strategic tracking

To quantify the extent to which participants adjusted their behavior in response to opponent threats, we estimated a lagged regression model for each participant.^12^ Specifically, we regressed a participant’s current contribution (C_j_; O_j_ for offender and D_j_ for defender) onto (i) the opponent group’s average contribution in the previous two rounds (G_j-1_+G_j-2_)/2, denoted as α, and (ii) the change in opponent contributions between the last two rounds (G_j-1_–G_j-2_), denoted as β:

For offenders:

$$O_{j}=\alpha \times \left(\frac{{\text{GD}}_{j-1\;}+{\text{GD}}_{j-2}}{2}\right)+\beta \times \left({\text{GD}}_{j-1}-\;{\text{GD}}_{j-2}\right)$$

For defenders:

$$D_{j}=\alpha \times \left(\frac{{\text{GO}}_{j-1\;}+{\text{GO}}_{j-2}}{2}\right)+\beta \times \left({\text{GO}}_{j-1}-\;{\text{GO}}_{j-2}\right)$$

The resulting standardized regression coefficient (β) captures the extent to which participants respond to changes in the opponent group’s behavior across rounds, serving as the index of strategic tracking. A positive β indicates a reactive strategy where investment scales with the perceived threat level (vigilance), whereas a negative β reflects a “strong-when-opponent-weaker” strategy (i.e., exploiting decreasing threat). A near-zero β indicates behavior independent of the opponent’s immediate history. All β coefficients were Fisher z-transformed before group-level analysis.

#### Performance and process metrics

We assessed performance and cognitive efficiency using four measures: (i) success rate: The proportion of rounds won (N_win_/24); (ii) earnings: the average monetary payoff across 24 rounds; (iii) decision rationality: Decision rationality was quantified as the negative absolute deviation between the observed contribution and the estimated best response (−|C_actual_ − C_best-response_|).^33^^,^^34^ Because participants could not observe current-round decisions by teammates or opponents at the time of choice, the best response was estimated from the most recent available information: teammates’ and opponents’ contributions in the immediately preceding round. For each participant and round, we identified the contribution that would maximize individual payoff assuming that others’ current-round contributions matched their previous-round contributions. Scores were averaged across rounds 2–24. Higher values indicate smaller deviations from the estimated best response and therefore more rational decisions; (iv) response time (RT): The latency between the onset of the decision screen and the participant’s response submission. To meet the assumptions of parametric analyses, raw RTs were log10-transformed to improve normality.^51^

#### Statistical analysis

All statistical analyses were conducted using IBM SPSS Statistics (version 27.0.1.0).

##### Behavioral analyses

We used 2 × 2 ANOVAs with factors Role (Offense, Defense) and Sex (Male, Female) to examine group differences in strategic indices and performance. Main and interaction effects of sex and role were tested. Following a significant Role × Sex interaction, we compared males and females within each role (Offense and Defense). *p*-values were adjusted for two comparisons using the Bonferroni procedure. These unadjusted ANOVA models constituted the primary behavioral analyses, as they directly tested the sex-by-role structure of behavior in the full behavioral sample.

Complementary sensitivity analyses were conducted using pooled single-slope ANCOVA models to account for variation in the opponent’s behavioral environment during repeated real-time interaction. Opponent average contribution was entered as a covariate, while Sex and Role were retained as between-subject factors. These models evaluated whether the primary sex- and role-related patterns remained evident after adjustment for average opponent contribution. As opponent average contribution was derived from dyadic task interaction rather than measured as a pre-existing participant characteristic, these covariate-adjusted models were interpreted as sensitivity analyses rather than replacements for the primary ANOVA models.

##### Associational analyses

Pearson correlation coefficients were calculated to examine associations between strategic indices and performance measures, and between strategic indices and testosterone measures. Fisher’s z-tests were used to compare the strength of these associations between roles. Testing-time-adjusted associations were evaluated using partial Pearson correlations controlling for testing time. Baseline-adjusted post-task associations were evaluated using partial Pearson correlations between post-task testosterone and the corresponding role-aligned behavioral strategy, controlling for pre-task testosterone. For the supplementary cross-time consistency analysis, testosterone values were residualized for assay plate, testing time, age, and behavioral role, followed by Spearman correlation analyses with bootstrap confidence intervals.

##### Statistical reporting

Unless otherwise specified, n denotes the number of participants contributing valid data to the corresponding analysis. Behavioral measures were calculated at the participant level using the rounds specified for each measure in STAR Methods. Exact sample sizes for individual analyses are reported in the Results, figure legends, and supplementary tables. Numerical group summaries are reported as mean ± SEM unless otherwise specified. For violin plots, medians and interquartile ranges (25th–75th percentiles) are shown in addition to means; whiskers extend to 1.5 × the interquartile range beyond the box and are truncated at the observed minimum or maximum. Confidence intervals are reported where indicated in the Results, figures, and supplementary materials. All statistical tests were two-tailed with α = 0.05. In figures, statistical significance is denoted as ∗*p* < 0.05, ∗∗*p* < 0.01, and ∗∗∗*p* < 0.001. Exact statistical tests, sample sizes, effect sizes, and other analysis-specific statistical details are reported in the corresponding Results, figure legends, and supplementary tables.

Figures were prepared using Python (version 3.8.20).
