# Supplementary material for: Offensive unpredictability and defensive tracking in intergroup conflict show sex-differentiated patterns
Source: iScience. 2026 Oct 15;29(11):117887. doi: 10.1016/j.isci.2026.117887 (PMC13694899; doi:10.1016/j.isci.2026.117887)
Supplement: Supplementary file 1 [file mmc1.pdf]

iScience, Volume *29*

Supplemental information

Offensive unpredictability and defensive tracking in intergroup conflict show sex-differentiated patterns

Hejing Zhang, Churuo Zhang, Anastasia Widjaja, and Yina Ma

Supplementary materials

Supplementary Figure 1 | Sex-stratified distributions of pre- and post-task assay-reported salivary testosterone values in the endocrine analytic sample.
Supplementary Figure 2 | Salivary testosterone covaries with role-aligned strategies in focal sex-by-role contexts.
Supplementary Figure 3 | Replication analysis in the endocrine analytic sample (n = 457): Offensive and defensive success rely on distinct behavioral strategies.
Supplementary Figure 4 | Replication analysis in the endocrine analytic sample (n = 457): Sex differences in role-specific strategies.
Supplementary Figure 5 | Replication analysis in the endocrine analytic sample (n = 457): Sex-differentiated performance advantages in offensive and defensive roles.

Supplementary Table 1 | Payoff matrix for the one-round IADC.
Supplementary Table 2 | Descriptive statistics for demographic characteristics.
Supplementary Table 3 | Descriptive statistics for assay-reported salivary testosterone values in the endocrine analytic sample (n = 457).
Supplementary Table 4 | Full statistics for role and sex effects on behavioral and performance measures in the full sample (n = 528).
Supplementary Table 5 | ANCOVA-based robustness analyses controlling for opponent's average contribution confirm the stability of behavioral effects (n = 528).
Supplementary Table 6 | Pearson Correlations, Means, and Standard Errors for Pre- and Post-task Testosterone (log10), Behavioral Variability, Strategic Tracking, and Success Rate by Role (Offensive vs. Defensive) and Sex
Supplementary Table 7 | Full statistics for role and sex effects on behavioral and performance measures in the endocrine analytic sample (n = 457).
Supplementary Table 8 | ANCOVA-based robustness analyses controlling for opponent's average contribution confirm the stability of behavioral effects (n = 457).
Supplementary Table 9 | ANCOVA-based robustness analysis controlling for testing time confirms stability of all behavioral effects.
Supplementary Table 10 | Testing-time-adjusted partial correlations between assay-reported salivary testosterone values (log10) and behavioral outcomes, stratified by sex and role.
Supplementary Table 11 | Cross-time consistency of pre-task and post-task assay-reported salivary testosterone values

Methods S1: Measurement scope and consistency of the exploratory endocrine analyses

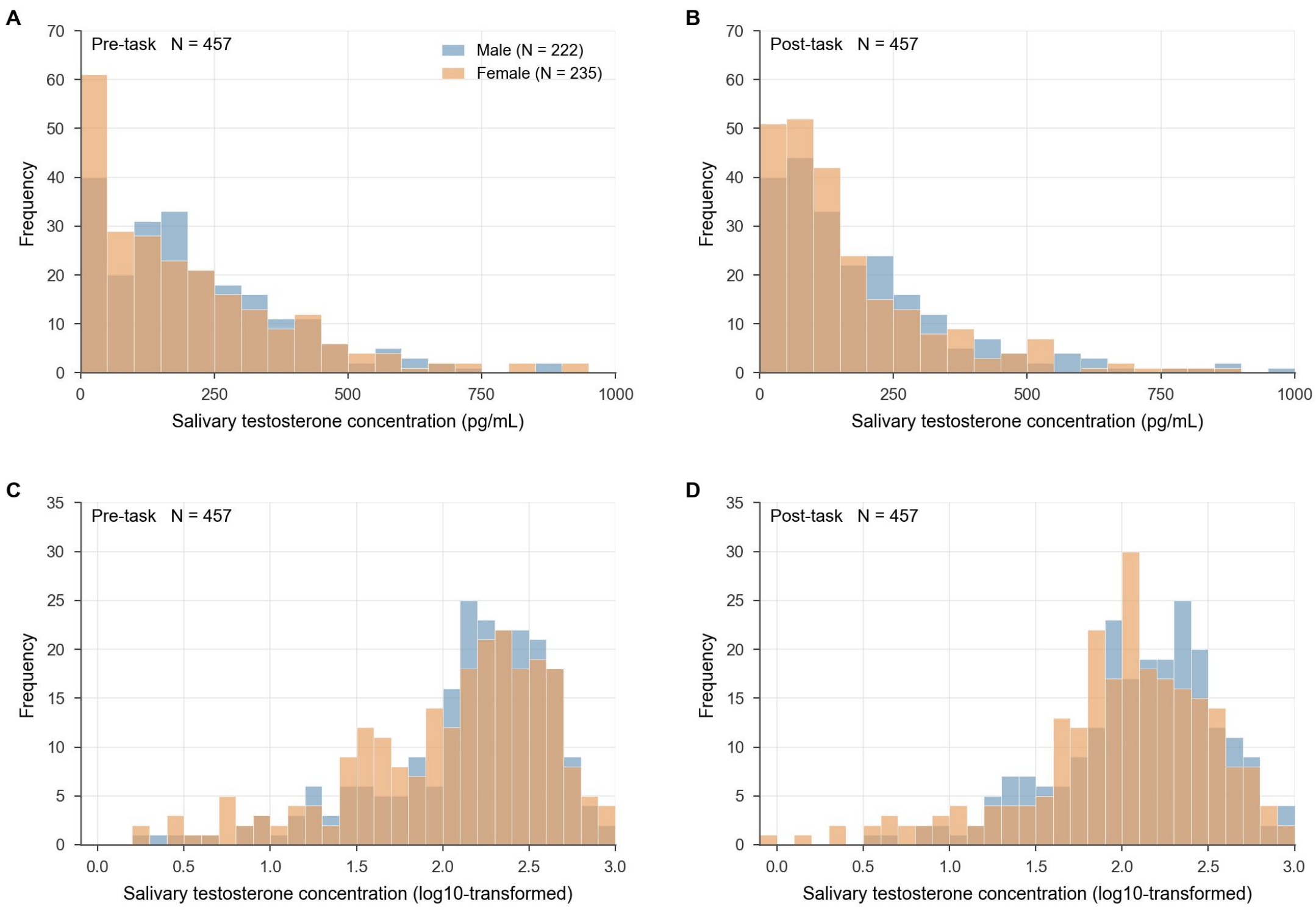

Supplementary Figure 1. Sex-stratified distributions of pre- and post-task assay-reported salivary testosterone values in the endocrine analytic sample. Panels A and B show raw pre-task and post-task values, respectively; Panels C and D show the corresponding log10-transformed values. Blue denotes men (n = 222) and orange denotes women (n = 235); the total endocrine analytic sample was n = 457. These distributions are descriptive and are not interpreted as validated estimates of absolute concentrations or biological between-sex endocrine differences.

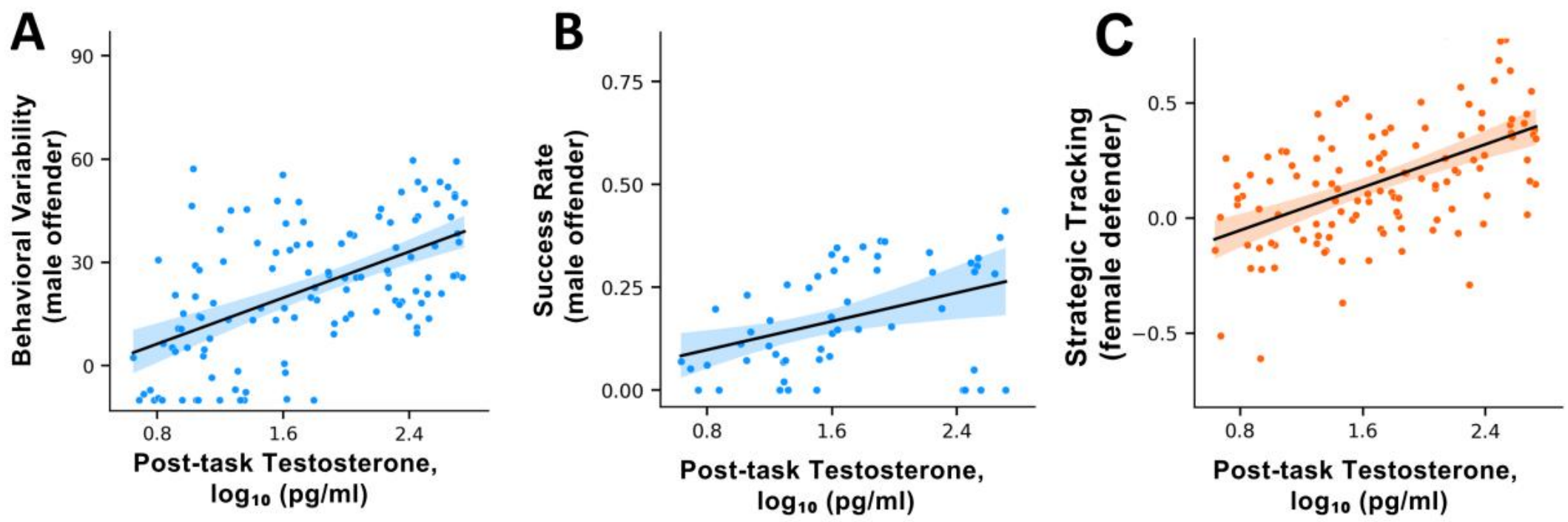

Supplementary Figure 2. Salivary testosterone covaries with role-aligned strategies in focal sex-by-role contexts. (A) In male offenders (n = 110), post-task log-transformed testosterone was positively associated with behavioral variability ($r_{108} = 0.287$, $p = 0.002$). **(B)** In the same subgroup, post-task log-transformed testosterone was positively associated with offensive success rate ($r_{108} = 0.207$, $p = 0.030$). **(C)** In female defenders (n = 105), post-task log-transformed testosterone was positively associated with strategic tracking ($r_{103} = 0.345$, $p < 0.001$). Each point represents one participant; the solid line indicates the linear fit and the shaded band indicates the 95% confidence interval.

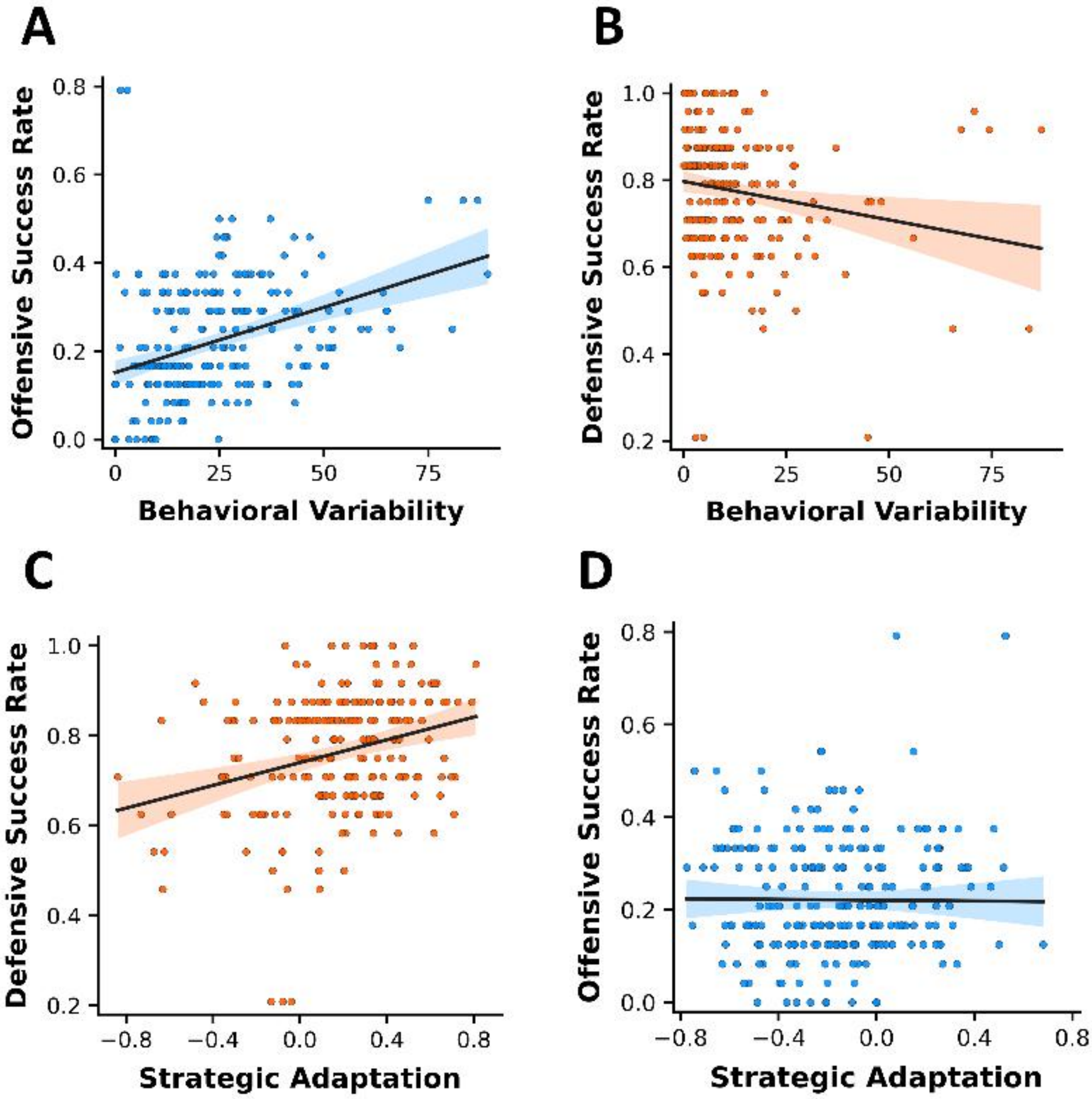

Supplementary Figure 3. Replication analysis in the endocrine analytic sample (n = 457): Offensive and defensive success rely on distinct behavioral strategies. **(A-B)** Behavioral variability was positively associated with success rate among offensive group (r_{232} = 0.379, $p < 0.001$), but negatively associated with success rate among defensive group (r_{221} = −0.174, p = 0.009). The difference between these two correlations was significant (Fisher's z = 6.100, p < 0.001). **(C-D)** Strategic tracking is not significantly associated with success rate among offensive group (r_{232} = −0.009, p = 0.897), but was positively associated with success rate among defensive group (r_{209} = 0.280, $p < 0.001$). The offensive–defensive difference was significant (Fisher's z = −3.104, p = 0.002). Each dot represents one participant (all measures aggregated across 24 rounds). Solid lines show linear fits; shaded bands indicate 95% confidence intervals.

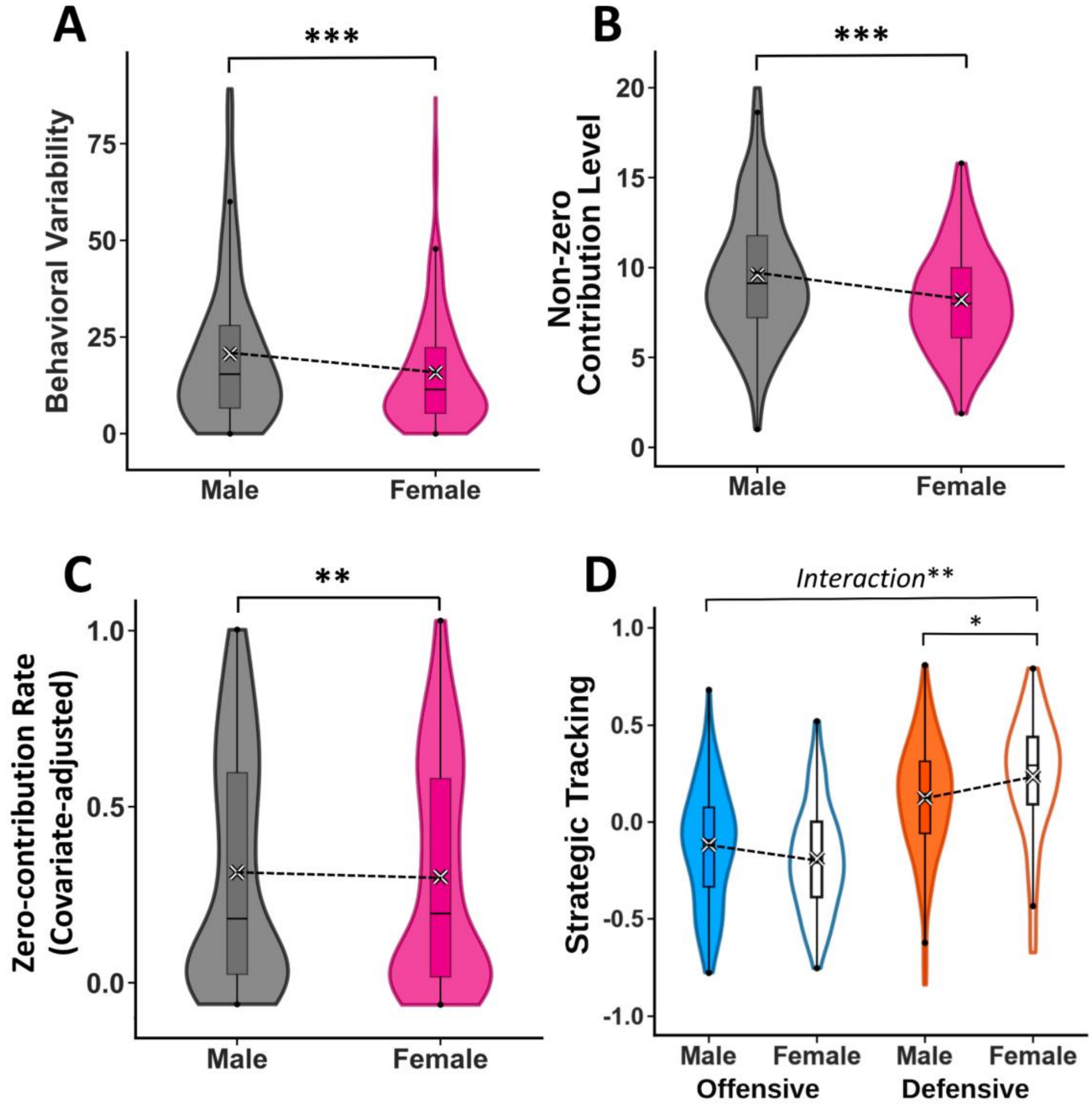

Supplementary Figure 4. Replication analysis in the endocrine analytic sample (n = 457): Sex differences in role-specific strategies. (A) Behavioral variability was higher in males than females (20.684 ± 1.069 vs. 15.522 ± 1.040; $F_{1,453} = 11.977$, $p < 0.001$, $\eta^2 = 0.026$). **(B)** Active-contribution magnitude was higher in males than females (9.625 ± 0.222 vs. 8.191 ± 0.216; $F_{1,439} = 21.448$, $p < 0.001$, $\eta^2 = 0.047$). **(C)** Zero-contribution frequency did not differ significantly between males and females (0.317 ± 0.015 vs. 0.286 ± 0.015; $F_{1,453} = 2.076$, p = 0.150, $\eta^2 = 0.005$). However, in the covariate-adjusted sensitivity analysis, males showed higher zero-contribution frequency than females after controlling for opponent's average contribution (adjusted means: 0.327 ± 0.014 vs. 0.275 ± 0.014; $F_{1,\ 452} = 7.048$, p = 0.008, $\eta^2 = 0.015$). **(D)** Strategic tracking differed significantly across the four sex × role groups (Interaction: $F_{1,441} = 10.279$, p = 0.001, $\eta^2 = 0.023$). Simple effects showed no significant sex difference among offensive group (−0.118 ± 0.028 vs. −0.189 ± 0.026; $F_{1,441} = 3.357$, $p_{corr} = 0.135$, $\eta^2 = 0.008$), whereas females in defensive group showed higher strategic tracking than males (0.233 ± 0.029 vs. 0.124 ± 0.029; $F_{1,441} = 7.194$, $p_{corr} = 0.015$, $\eta^2 = 0.016$). Violin plots show the distributions. Horizontal lines indicate medians, crosses indicate means, and whiskers extend to 1.5 × the interquartile range beyond the box and are truncated at the observed minimum or maximum. Numerical values are group

means ± SEM. *$p < 0.05$, **$p < 0.01$, ***$p < 0.001$.

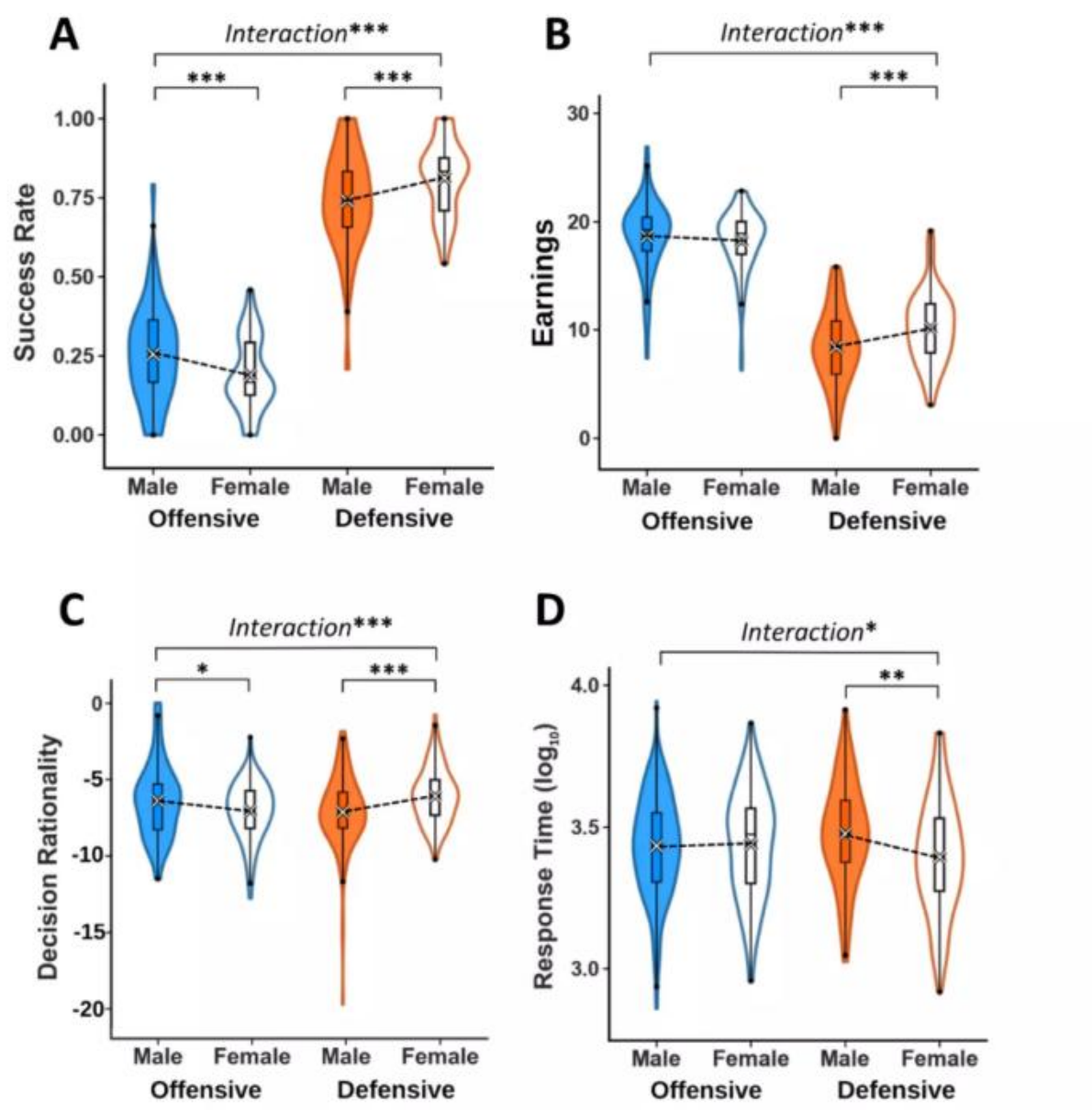

Supplementary Figure 5. Replication analysis in the endocrine analytic sample (n = 457): Sex-differentiated performance advantages in offensive and defensive roles. (A) Success rate showed a significant sex × role interaction ($F_{1,453} = 28.586$, $p < 0.001$, $\eta_p^2 = 0.059$). Simple effects showed that males in the offensive group had higher success rates than female offenders (0.256 ± 0.015 vs. 0.191 ± 0.010; $F_{1,453} = 13.210$, $p_{corr} < 0.001$, $\eta_p^2 = 0.028$), whereas females had higher success rates than males in the defensive group (0.812 ± 0.011 vs. 0.739 ± 0.015; $F_{1,453} = 15.398$, $p_{corr} < 0.001$, $\eta_p^2 = 0.033$). **(B)** Earnings showed a significant sex × role interaction ($F_{1,453} = 12.920$, $p < 0.001$, $\eta_p^2 = 0.028$). Simple effects showed no significant sex difference in the offensive group (18.621 ± 0.321 vs. 18.190 ± 0.263; $F_{1,453} = 1.024$, $p_{corr} = 0.624$, $\eta_p^2 = 0.002$), whereas females received higher payments than males in the defensive group (10.166 ± 0.309 vs. 8.410 ± 0.325; $F_{1,453} = 16.306$, $p_{corr} < 0.001$, $\eta_p^2 = 0.035$). **(C)** Decision rationality showed a significant sex × role interaction ($F_{1,453} = 18.551$, $p < 0.001$, $\eta_p^2 = 0.039$). Simple effects showed that females in the offensive group had higher error than male offenders (-7.087 ± 0.170 vs. -6.395 ± 0.234; $F_{1,453} = 5.892$, $p_{corr} = 0.031$, $\eta_p^2 = 0.013$), whereas males had higher error than females in the defensive group (-7.147 ± 0.234 vs. -6.083 ± 0.173; $F_{1,453} = 13.326$, $p_{corr} < 0.001$, $\eta_p^2 = 0.029$). **(D)** Response time showed a significant sex × role interaction ($F_{1,453} = 6.614$, $p = 0.010$, $\eta_p^2 = 0.014$). Simple effects showed no significant sex difference in the offensive group (3.434 ± 0.017 vs. 3.440 ± 0.017; $F_{1,453} = 0.064$, $p_{corr} = 1.000$, $\eta_p^2 < 0.001$), whereas males had longer response times than females in the defensive group (3.478 ± 0.017 vs. 3.394 ± 0.019; $F_{1,453} = 11.220$, $p_{corr} = 0.002$, $\eta_p^2 = 0.024$). Violin plots show the distributions. Horizontal lines indicate medians, crosses indicate means, and whiskers extend to 1.5 × the interquartile range beyond the box and are truncated at the observed minimum or maximum. Numerical values are group means ± SEM. *$p < 0.05$, **$p < 0.01$, ***$p < 0.001$.

Supplementary Table 1 | Payoff matrix of one-round IADC

Role	Participant	Initial endowment	Individual contribution (Ii)	Group pool (G)	Payment		
					Offenders lose GO ≤ GD	Offenders win GO > GD	
					Leftover	Leftover	Spoil
Offender	Offender1	20	IO1	GO	20 - IO1	20 - IO1	(60 - GD)/3
	Offender2	20	IO2		20 - IO2	20 - IO2	(60 - GD)/3
	Offender3	20	IO3		20 - IO3	20 - IO3	(60 - GD)/3
Defender	Defender1	20	ID1	GD	20 - ID1	0	0
	Defender2	20	ID2		20 - ID2	0	0
	Defender3	20	ID3		20 - ID3	0	0

Note. Each participant received an endowment of 20 tokens in each round. Individual payoff depended on the comparison between the offender group pool (GO) and defender group pool (GD). If GO> GD, offenders retained their own uninvested tokens and equally shared the defenders' remaining uninvested tokens, whereas defenders received 0. If GO ≤ GD, all participants retained only their own uninvested tokens.

Supplementary Table 2 | Descriptive statistics for participants' demographic characteristics

A. Full behavioral sample (n = 528)

Group	N	Age		Education	
		Mean ± SEM	95% CI	Mean ± SEM	95% CI
Male offenders	120	22.60 ± 0.26	[22.09, 23.11]	17.17 ± 0.16	[16.85, 17.49]
Female offenders	144	21.76 ± 0.20	[21.36, 22.15]	16.93 ± 0.13	[16.68, 17.19]
Male defenders	120	22.84 ± 0.25	[22.35, 23.33]	17.10 ± 0.17	[16.77, 17.43]
Female defenders	144	21.42 ± 0.20	[21.03, 21.81]	16.78 ± 0.14	[16.50, 17.06]
Total	528	22.10 ± 0.11	[21.88, 22.33]	16.98 ± 0.07	[16.84, 17.13]

Note. Age is reported in years. Education is reported as years of education. Values are presented as mean ± SEM and 95% confidence intervals (CIs). CIs were calculated using the t distribution. Education data were missing for three male offenders; therefore, Education statistics for male offenders are based on n = 117 and total Education statistics are based on n = 525.

B. Endocrine analytic sample (n = 457)

Group	N	Age		Education	
		Mean ± SEM	95% CI	Mean ± SEM	95% CI
Male offenders	110	22.56 ± 0.27	[22.02, 23.11]	17.10 ± 0.16	[16.78, 17.43]
Female offenders	124	21.73 ± 0.21	[21.31, 22.14]	16.98 ± 0.14	[16.70, 17.27]
Male defenders	112	22.90 ± 0.26	[22.38, 23.42]	17.10 ± 0.17	[16.76, 17.44]
Female defenders	111	21.46 ± 0.23	[21.00, 21.92]	16.69 ± 0.15	[16.39, 17.00]
Total	457	22.15 ± 0.12	[21.91, 22.40]	16.97 ± 0.08	[16.81, 17.12]

Note. Age is reported in years. Education is reported as years of education. Values are presented as mean ± SEM and 95% confidence intervals (CIs). CIs were calculated using the t distribution. Education data were missing for two male offenders; therefore, Education statistics for male offenders are based on n = 108 and total Education statistics are based on n = 455.

Supplementary Table 3 | Descriptive statistics for assay-reported salivary testosterone values in the endocrine analytic sample (n = 457).

Group	N	Pre-task mean ± SEM	Pre-task median [IQR]	Pre-task range	Post-task mean ± SEM	Post-task median [IQR]	Post-task range
Male offenders	110	207.91 ± 14.04	188.98 [86.44–316.76]	2.35–640.83	194.27 ± 17.51	146.23 [60.44–250.20]	3.31–963.34
Female offenders	124	193.97 ± 16.49	145.91 [48.99–275.23]	1.90–938.63	163.89 ± 13.92	114.40 [61.96–214.56]	0.96–896.19
Male defenders	112	222.88 ± 18.03	171.51 [87.74–304.76]	1.63–852.63	190.96 ± 16.11	137.22 [75.06–253.40]	7.73–873.37
Female defenders	111	207.43 ± 18.34	143.27 [43.26–324.36]	2.62–837.48	168.82 ± 16.18	109.84 [52.75–237.91]	2.10–812.01
Male participants	222	215.46 ± 11.44	176.10 [85.54–310.49]	1.63–852.63	192.60 ± 11.86	142.11 [74.72–252.58]	3.31–963.34
Female participants	235	200.33 ± 12.26	143.27 [46.33–288.70]	1.90–938.63	166.22 ± 10.58	113.28 [55.07–232.10]	0.96–896.19
Total	457	207.68 ± 8.40	165.31 [64.00–308.68]	1.63–938.63	179.03 ± 7.94	122.04 [63.88–243.09]	0.96–963.34

Note. Values are assay-reported outputs (pg/mL) from cotton-swab samples quantified using the DRG Salivary Testosterone ELISA kit. The manufacturer-specified analytical range was used to define the endocrine analytic sample (0.94-1000 pg/mL). Both the mean ± SEM and the median [IQR] are reported as complementary summaries of the data; the median [IQR] is particularly informative for the right-skewed raw distributions. SEM, standard error; IQR, interquartile range.

Supplementary Table 4 | Full statistics for role- and sex-effects across behavioral and performance measures (N=528).

a. Descriptive statistics for all measures

Index	Offender		Defender	
	Male	Female	Male	Female
Behavioral Variability	26.972 (1.912)	20.640 (1.173)	14.187 (1.256)	10.381 (1.109)
Zero-contribution Rate	0.529 (0.027)	0.510 (0.024)	0.094 (0.016)	0.053 (0.010)
Non-zero Contribution Level	10.208 (0.345)	8.548 (0.258)	9.163 (0.311)	7.996 (0.242)
Strategic Tracking	-0.107 (0.028)	-0.200 (0.023)	0.120 (0.027)	0.223 (0.025)
Success Rate	0.259 (0.014)	0.189 (0.009)	0.741 (0.014)	0.811 (0.009)
Payment	18.281 (0.339)	18.159 (0.246)	8.485 (0.309)	9.985 (0.273)
Decision Rationality	-6.433 (0.234)	-7.124 (0.154)	-7.077 (0.223)	-6.257 (0.158)
Response Time (log10)	3.441 (0.017)	3.430 (0.016)	3.481 (0.016)	3.405 (0.016)

b. Two-way ANOVA results for all measures

Index	Gender			Role			Interaction		
	F	p	η2	F	p	η2	F	p	η2
Behavioral Variability	13.731	<0.001	0.026	70.941	<0.001	0.119	0.852	0.356	0.002
Zero-contribution Rate	2.299	0.130	0.004	489.739	<0.001	0.483	0.310	0.578	0.001
Non-zero Contribution Level	24.277	<0.001	0.046	7.751	0.006	0.015	0.738	0.391	0.001
Strategic Tracking	0.047	0.828	0.000	158.209	<0.001	0.236	14.415	<0.001	0.027
Success Rate	0.000	1.000	0.000	2169.390	<0.001	0.805	35.103	<0.001	0.063
Payment	5.637	0.018	0.011	958.973	<0.001	0.647	7.806	0.005	0.015
Decision Rationality	0.115	0.735	0.000	0.343	0.559	0.001	15.751	<0.001	0.029
Response Time (log10)	7.256	0.007	0.014	0.225	0.635	0.000	3.974	0.047	0.008

Supplementary Table 5 | ANCOVA-based robustness analyses controlling for opponent's average contribution confirm the stability of behavioral effects (n = 528).

A. Estimated marginal means for all measures

Index	Offender		Defender	
	Male	Female	Male	Female
Behavioral Variability	24.377(1.463)	18.904(1.305)	15.846(1.420)	12.897(1.344)
Zero-contribution Rate	0.598(0.020)	0.556(0.018)	0.050(0.019)	-0.014(0.018)
Non-zero Contribution Level	8.832(0.257)	7.501(0.231)	10.044(0.242)	9.323(0.229)
Strategic Tracking	-0.102(0.028)	-0.197(0.025)	0.117(0.028)	0.218(0.026)
Success Rate	0.281(0.013)	0.204(0.011)	0.727(0.012)	0.790(0.012)
Payment	20.189(0.179)	19.436(0.159)	7.265(0.173)	8.134(0.164)
Response Time (log10)	3.417(0.018)	3.414(0.016)	3.497(0.017)	3.428(0.016)
Decision Rationality	-6.427(0.210)	-7.120(0.187)	-7.081(0.203)	-6.263(0.193)

B. Effect tests for all measures

Index	Opponent's average contribution			Gender			Role			Interaction		
	F	p	η^2	F	p	η^2	F	p	η^2	F	p	η^2
Behavioral Variability	31.720	<0.001	0.057	9.902	0.002	0.019	22.596	<0.001	0.041	0.901	0.343	0.002
Zero-contribution Rate	119.057	<0.001	0.185	8.466	0.004	0.016	712.315	<0.001	0.577	0.379	0.538	0.001
Non-zero Contribution Level	285.290	<0.001	0.360	19.748	<0.001	0.037	32.152	<0.001	0.060	1.753	0.186	0.003
Strategic Tracking	0.330	0.566	0.001	0.021	0.886	0.000	116.644	<0.001	0.186	14.353	<0.001	0.027
Success Rate	29.437	<0.001	0.053	0.381	0.537	0.001	1513.514	<0.001	0.743	37.015	<0.001	0.066
Payment	1148.680	<0.001	0.687	0.126	0.723	0.000	4203.393	<0.001	0.889	24.940	<0.001	0.046
Response Time (log10)	18.605	<0.001	0.034	4.972	0.026	0.009	6.502	0.011	0.012	4.109	0.043	0.008
Decision Rationality	0.009	0.923	0.000	0.106	0.745	0.000	0.212	0.645	0.000	15.721	<0.001	0.029

Supplementary Table 6 | Pearson Correlations, Means, and Standard Errors for Pre- and Post-task Testosterone (log10), Behavioral Variability, Strategic Tracking, and Success Rate by Role (Offensive vs. Defensive) and Sex

A. Offensive male, n = 110

Index	1	2	3	4	5	M	SE
1. Pre-task testosterone (log10)	—					2.136	0.047
2. Post-task testosterone (log10)	0.628*	—				2.083	0.045
3. Behavioral variability	0.225*	0.287*	—			27.241	1.996
4. Strategic tracking	0.107	0.105	0.061	—		-0.118	0.029
5. Success rate	0.067	0.207*	0.350*	-0.026	—	0.256	0.015

B. Defensive male, n = 112

Index	1	2	3	4	5	M	SE
1. Pre-task testosterone (log10)	—					2.130	0.051
2. Post-task testosterone (log10)	0.624*	—				2.098	0.042
3. Behavioral variability	-0.060	-0.008	—			14.127	1.322
4. Strategic tracking	0.133	0.133	-0.272*	—		0.124	0.029
5. Success rate	-0.088	-0.057	-0.336*	0.301*	—	0.739	0.015

C.Offensive female, n = 124

Index	1	2	3	4	5	M	SE
1. Pre-task testosterone (log10)	—					2.040	0.050
2. Post-task testosterone (log10)	0.544*	—				1.995	0.047
3. Behavioral variability	0.166	0.072	—			20.379	1.213
4. Strategic tracking	-0.014	0.055	0.017	—		-0.189	0.025
5. Success rate	-0.009	-0.082	0.350*	-0.057	—	0.191	0.010

D.Defensive female, n = 111

Index	1	2	3	4	5	M	SE
1. Pre-task testosterone (log10)	—					2.040	0.057
2. Post-task testosterone (log10)	0.751*	—				1.969	0.053
3. Behavioral variability	0.048	0.045	—			10.665	1.370
4. Strategic tracking	0.224*	0.345*	-0.114	—		0.233	0.030
5. Success rate	0.132	0.137	0.093	0.179	—	0.812	0.011

E. Baseline-adjusted focal post-task associations

**Subgroup**	**Behavioral outcome**	**Partial *r***	***p***
Offensive male	Behavioral variability	0.192	0.046
Defensive female	Strategic tracking	0.275	0.005

F. Between-role comparisons of focal testosterone–strategy associations

**Sex**	**Focal association**	**Role comparison**	**Fisher's *z***	***p***
Male	Post-task testosterone–behavioral variability	Offensive vs. defensive	2.23	0.026
Female	Post-task testosterone–strategic tracking	Defensive vs. offensive	2.26	0.024

Note. Association analyses used log10-transformed assay-reported salivary testosterone values. Panels A–D report Pearson correlation coefficients (lower triangle), means (M), and standard errors (SE), with pre-task and post-task testosterone associations examined separately. Panel E reports partial correlations between post-task testosterone and the corresponding role-aligned behavioral strategy after controlling for pre-task testosterone. Panel F reports Fisher's z tests comparing the focal post-task testosterone–strategy correlations between behavioral roles within each sex. Correlations used pairwise complete observations. Strategic tracking was available for n = 106 male defenders and n = 105 female defenders; all other variables used the full subgroup N. $^*p < 0.05$.

Supplementary Table 7 | Full statistics for role and sex effects on behavioral and performance measures in the endocrine analytic sample (n = 457).

A. Estimated marginal means for all measures

Index	Offender		Defender	
	Male	Female	Male	Female
Behavioral Variability	27.241(1.518)	20.379(1.430)	14.127(1.505)	10.665(1.511)
Zero-contribution Rate	0.547(0.022)	0.511(0.021)	0.087(0.022)	0.062(0.022)
Non-zero Contribution Level	10.054(0.321)	8.542(0.301)	9.195(0.308)	7.839(0.309)
Strategic Tracking	-0.118(0.028)	-0.189(0.026)	0.124(0.029)	0.233(0.029)
Success Rate	0.256(0.013)	0.191(0.012)	0.739(0.013)	0.812(0.013)
Payment	18.621(0.310)	18.190(0.292)	8.410(0.307)	10.166(0.308)
Response Time (log10)	3.434(0.018)	3.440(0.017)	3.478(0.018)	3.394(0.018)
Decision Rationality	-6.395(0.207)	-7.087(0.195)	-7.147(0.206)	-6.083(0.206)

B. Effect tests for all measures

Index	Gender			Role			Interaction		
	F	p	η^2	F	p	η^2	F	p	η^2
Behavioral Variability	11.977	<0.001	0.026	58.562	<0.001	0.114	1.300	0.255	0.003
Zero-contribution Rate	2.076	0.150	0.005	450.089	<0.001	0.498	0.055	0.815	0.000
Non-zero Contribution Level	21.448	<0.001	0.047	6.354	0.012	0.014	0.063	0.802	0.000
Strategic Tracking	0.463	0.497	0.001	140.377	<0.001	0.241	10.279	0.001	0.023
Success Rate	0.070	0.792	0.000	1833.167	<0.001	0.802	28.586	<0.001	0.059
Payment	4.750	0.030	0.010	898.900	<0.001	0.665	12.920	<0.001	0.028
Response Time (log10)	4.919	0.027	0.011	0.003	0.956	0.000	6.614	0.010	0.014
Decision Rationality	0.833	0.362	0.002	0.384	0.536	0.001	18.551	<0.001	0.039

Supplementary Table 8 | ANCOVA-based robustness analyses controlling for opponent's average contribution confirm the stability of behavioral effects (n = 457).

A. Estimated marginal means for all measures (adjusted at opponent's average contribution=mean)

Index	Offender		Defender	
	Male	Female	Male	Female
Behavioral Variability	24.736(1.536)	18.463(1.425)	15.835(1.488)	13.565(1.551)
Non-zero Contribution Level	8.867(0.274)	7.534(0.255)	9.999(0.257)	9.193(0.268)
Zero-contribution Rate	0.606(0.021)	0.556(0.019)	0.047(0.020)	-0.007(0.021)
Strategic Tracking	-0.113(0.029)	-0.185(0.027)	0.120(0.029)	0.226(0.030)
Success Rate	0.277(0.013)	0.206(0.012)	0.725(0.013)	0.788(0.013)
Payment	20.280(0.181)	19.459(0.168)	7.278(0.175)	8.246(0.183)
Response Time (log10)	3.413(0.018)	3.424(0.017)	3.492(0.018)	3.418(0.019)
Decision Rationality	-6.429(0.217)	-7.113(0.201)	-7.124(0.210)	-6.044(0.219)

B. Effect tests for all measures

Index	opponent's average contribution			Gender			Role			Interaction		
	F	p	η^2	F	p	η^2	F	p	η^2	F	p	η^2
Behavioral Variability	31.580	<0.001	0.065	8.653	0.003	0.019	17.441	<0.001	0.037	1.919	0.167	0.004
Non-zero Contribution Level	220.771	<0.001	0.335	17.719	<0.001	0.039	22.818	<0.001	0.050	1.086	0.298	0.002
Zero-contribution Rate	97.557	<0.001	0.178	7.048	0.008	0.015	636.118	<0.001	0.585	0.012	0.914	0.000
Strategic Tracking	0.392	0.532	0.001	0.365	0.546	0.001	104.043	<0.001	0.191	10.082	0.002	0.022
Success Rate	27.967	<0.001	0.058	0.094	0.759	0.000	1291.568	<0.001	0.741	28.130	<0.001	0.059
Payment	1000.296	<0.001	0.689	0.185	0.667	0.000	3879.780	<0.001	0.896	27.665	<0.001	0.058
Response Time (log10)	14.705	<0.001	0.032	3.311	0.069	0.007	3.296	0.070	0.007	6.084	0.014	0.013
Decision Rationality	0.282	0.595	0.001	0.930	0.335	0.002	0.639	0.424	0.001	18.666	<0.001	0.040

Supplementary Table 9 | ANCOVA-based robustness analysis controlling for Testing time confirms stability of all behavioral effects.

A. Estimated marginal means for all measures

Index	Offender		Defender	
	Male	Female	Male	Female
Behavioral Variability	26.947(1.430)	20.661(1.306)	14.162(1.430)	10.402(1.306)
Zero-contribution Rate	0.527(0.021)	0.511(0.019)	0.093(0.021)	0.054(0.019)
Non-zero Contribution Level	10.210(0.304)	8.546(0.279)	9.165(0.295)	7.995(0.270)
Strategic Tracking	-0.107(0.027)	-0.200(0.024)	0.120(0.027)	0.222(0.025)
Success Rate	0.259(0.012)	0.189(0.011)	0.741(0.012)	0.811(0.011)
Payment	18.261(0.302)	18.176(0.276)	8.465(0.302)	10.002(0.276)
Response Time (log10)	3.442(0.017)	3.429(0.016)	3.482(0.017)	3.404(0.016)
Decision Rationality	-6.430(0.199)	-7.127(0.182)	-7.074(0.199)	-6.260(0.182)

B. **Effect tests for all measures**

Index	Testing time			Gender			Role			Interaction		
	F	p	η^2	F	p	η^2	F	p	η^2	F	p	η^2
Behavioral Variability	0.343	0.558	0.001	13.425	<0.001	0.025	70.852	<0.001	0.119	0.851	0.357	0.002
Zero-contribution Rate	9.673	0.002	0.018	1.822	0.178	0.003	497.845	<0.001	0.488	0.316	0.575	0.001
Non-zero Contribution Level	0.031	0.860	0.000	24.236	<0.001	0.046	7.726	0.006	0.015	0.741	0.390	0.001
Strategic Tracking	0.502	0.479	0.001	0.028	0.867	0.000	158.028	<0.001	0.237	14.331	<0.001	0.027
Success Rate	0.000	1.000	0.000	0.000	1.000	0.000	2165.250	<0.001	0.805	35.036	<0.001	0.063
Payment	5.188	0.023	0.010	6.295	0.012	0.012	966.636	<0.001	0.649	7.869	0.005	0.015
Response Time (log10)	2.366	0.125	0.005	7.730	0.006	0.015	0.226	0.635	0.000	3.984	0.046	0.008
Decision Rationality	0.336	0.563	0.001	0.093	0.761	0.000	0.342	0.559	0.001	15.731	<0.001	0.029

Supplementary Table 10 | Testing-time-adjusted partial correlations between assay-reported salivary testosterone values (log10) and behavioral outcomes, stratified by sex and role.

A. Offensive male, n = 110

Index	Pre-task partial r	Post-task partial r	M	SE
1. Pre-task testosterone (log10)	—	—	2.136	0.047
2. Post-task testosterone (log10)	—	—	2.083	0.045
3. Behavioral variability	0.218*	0.286*	27.241	1.996
4. Strategic tracking	0.109	0.106	-0.118	0.029
5. Success rate	0.042	0.208*	0.256	0.015

B. Defensive male, n = 112

Index	Pre-task partial r	Post-task partial r	M	SE
1. Pre-task testosterone (log10)	—	—	2.130	0.051
2. Post-task testosterone (log10)	—	—	2.098	0.042
3. Behavioral variability	-0.069	-0.015	14.127	1.322
4. Strategic tracking	0.138	0.133	0.124	0.029
5. Success rate	-0.070	-0.041	0.739	0.015

C. Offensive female, n = 124

Index	Pre-task partial r	Post-task partial r	M	SE
1. Pre-task testosterone (log10)	—	—	2.040	0.050
2. Post-task testosterone (log10)	—	—	1.995	0.047
3. Behavioral variability	0.170	0.074	20.379	1.213
4. Strategic tracking	-0.033	0.044	-0.189	0.025
5. Success rate	-0.019	-0.089	0.191	0.010

D. Defensive female, n = 111

Index	Pre-task partial r	Post-task partial r	M	SE
1. Pre-task testosterone (log10)	—	—	2.040	0.057
2. Post-task testosterone (log10)	—	—	1.969	0.053
3. Behavioral variability	0.008	0.013	10.665	1.370
4. Strategic tracking	0.230*	0.352*	0.233	0.030
5. Success rate	0.165	0.162	0.812	0.011

Note. The Pre-task and Post-task columns report partial correlations between log10-transformed assay-reported values and the row measure, controlling for testing time. M and SE are shown for each measure. Strategic tracking was available for n = 106 male defenders and n = 105 female defenders; all other measures used the full subgroup N. *p < 0.05.

Supplementary Table 11 | Cross-time consistency of pre-task and post-task assay-reported salivary testosterone values

Analysis	Sample	*N*	Correlation coefficient	95% CI	*p*
Pearson correlation	Full endocrine sample	457	$r = 0.641$	[0.583, 0.692]	< 0.001
Pearson correlation	Male participants	222	$r = 0.624$	[0.536, 0.698]	< 0.001
Pearson correlation	Female participants	235	$r = 0.648$	[0.567, 0.717]	< 0.001
Spearman correlation	Male participants	222	$\rho = 0.630$	[0.529, 0.716]	< 0.001
Spearman correlation	Female participants	235	$\rho = 0.639$	[0.548, 0.715]	< 0.001
Residualized Spearman correlation	Male participants	222	$\rho = 0.398$	[0.279, 0.508]	< 0.001
Residualized Spearman correlation	Female participants	235	$\rho = 0.526$	[0.419, 0.621]	< 0.001

Note. Analyses used paired log10-transformed assay-reported salivary testosterone values. Pre-task and post-task values were separately residualized in the full endocrine sample (n = 457) for the corresponding assay plate, testing time, age, and behavioral role before within-sex Spearman correlations were calculated. Pearson confidence intervals used Fisher's r-to-z transformation; all Spearman confidence intervals used 100,000 paired percentile bootstrap resamples within each sex, with fitted residuals held fixed for residualized analyses. All p values are two-sided, with asymptotic p values reported for Spearman correlations. CI, confidence interval.

Methods S1: Measurement scope and consistency of the exploratory endocrine analyses

All endocrine analyses used the endocrine analytic sample (n = 457; 222 male and 235 female participants, Table S2B). This sample comprised participants with paired pre-task and post-task assay-reported salivary testosterone values within the manufacturer-specified analytical range of the DRG Salivary Testosterone ELISA kit (0.94–1000 pg/mL).

The raw assay-reported values were right-skewed. Accordingly, Table S3 reports means, standard errors, medians, interquartile ranges, and observed ranges for the raw assay-reported values, and Figure S1 displays the corresponding raw and log10-transformed distributions.

Published work indicates that both saliva-collection procedures and analytical platforms can affect testosterone estimates. Salivette-based and passive-drool measurements differ in their correspondence with serum free testosterone.[1] In a large study using cotton Salivettes and ELISA, salivary testosterone showed a markedly attenuated apparent sex difference but retained modest associations with plasma testosterone within each sex.[2] Cross-platform comparisons further indicate that immunoassays can inflate estimates at lower testosterone concentrations, particularly in women,[3] and that absolute estimates vary across analytical platforms and laboratories.[4] Accordingly, we refer to the present measurements as assay-reported salivary testosterone values rather than analytically validated estimates of absolute salivary or circulating testosterone concentrations. The inferential focus was therefore relative between-person variation within sex. Absolute circulating concentrations and the magnitude of between-sex endocrine differences were outside the scope of these analyses.

Pre-task and post-task samples were assayed on different plates, allowing cross-time consistency to be evaluated independently of a shared plate. Cross-time consistency was assessed using Pearson and Spearman correlations between pre-task and post-task log10-transformed assay-reported values. To assess the robustness of this consistency to measured procedural and participant characteristics, pre-task and post-task values were separately residualized for assay plate, testing time, age, and behavioral role. Within-sex rank-order consistency of the residualized values was then assessed using Spearman correlations with bootstrap confidence intervals. Full results of the cross-time consistency analyses are reported in Table S11.

Supplemental References:

1. Fiers, T. *et al.* A critical evaluation of salivary testosterone as a method for the assessment of serum testosterone. *Steroids* **86**, 5–9 (2014).
2. De Wit, A. E. *et al.* Testosterone in human studies: Modest associations between plasma and salivary measurements. *Andrologia* **50**, e12779 (2018).
3. Welker, K. M. *et al.* A comparison of salivary testosterone measurement using immunoassays and tandem mass spectrometry. *Psychoneuroendocrinology* **71**, 180–188 (2016).
4. Dlugash, G. *et al.* Multicenter comparison of LC-MS/MS, radioimmunoassay, and ELISA for assessment of salivary cortisol and testosterone. *Psychoneuroendocrinology* **183**, 107618 (2026).
